# Correction: Novel comparison of evaluation metrics for gene ontology classifiers reveals drastic performance differences

**DOI:** 10.1371/journal.pcbi.1010249

**Published:** 2022-06-09

**Authors:** Ilya Plyusnin, Liisa Holm, Petri Törönen

There are several errors in Table 1. The values for the column rec in rows ic SimGIC2, ic2 SimGIC2, AJacc E, and ic2 Smin1 are incorrect. Please see the correct Table 1 here.

**Table 1:** Summary of results for best performing and widely-used metrics. Here we show RC (Rank Correlation) and FP (False Positive) results for the best performing methods. We also show same results for some widely-used metrics. Good metrics should have a high RC score and low FP scores. Rec column shows our selected recommendations (See text for details). The five best results in each column are shown in bold. The five weakest results in each column are shown with underlined italics. Metrics that fail a given test are highlighted in red (see text for details). Note how methods in lower block show consistent weak performance either in RC or FP tests.

Top performing metrics								
	Rank Correlation Results			False Positive Sets				
Metric	UniProt	CAFA	Mouse	UniProt	CAFA	Mouse	rec	weakness
TC AUCROC	0.959	0.951	*0*.*920*	**0.023**	**0.010**	**0.000**	(*)	RC in mouse data
TC AUCPR	**0.984**	**0.982**	0.971	0.144	0.156	*0*.*312*	(*)	FPS in mouse data
ic SimGIC	0.960	0.963	0.969	0.168	0.164	0.108		
ic SimGIC2	0.970	0.969	0.965	0.166	0.133	**0.056**	*	
ic2 SimGIC2	**0.979**	0.978	0.974	0.190	0.156	0.065	(*)	
Resnik E	0.966	0.959	0.945	**0.034**	**0.064**	0.065	(*)	RC in mouse data
Lin E	0.939	**0.983**	**0.982**	0.112	0.118	0.080	(*)	
Lin F	*0*.*856*	0.979	**0.978**	**0.096**	**0.100**	**0.058**		very weak RC in Uniprot
Ajacc E	*0*.*886*	0.960	0.959	0.097	0.127	0.073		very weak RC in Uniprot
ic2 S_*min1*_	**0.986**	**0.985**	**0.983**	0.247	0.221	0.124	(*)	Slightly weak in FPS
Previously used metrics with weaker performance								
F_*max*_	**0.983**	**0.982**	**0.981**	0.367	0.318	0.229		weak in FPS tests
US AUCPR	**0.985**	**0.983**	**0.977**	*0*.*453*	*0*.*388*	*0*.*292*		weak in FPS tests
US AUCROC	0.945	*0*.*932*	*0*.*901*	*1*.*000*	*1*.*000*	*0*.*878*		worst metrics in FPS tests
GC AUCROC	0.947	0.937	0.921	*1*.*000*	*1*.*000*	*0*.*879*		worst metrics in FPS tests
Resnik A	0.922	*0*.*808*	*0*.*772*	**0.000**	**0.000**	**0.000**		weak in RC tests
Resnik D	*0*.*892*	*0*.*811*	*0*.*801*	*0*.*428*	*0*.*333*	*0*.*339*		weak in all tests
Lin A	*0*.*758*	*0*.*840*	*0*.*880*	**0.000**	**0.000**	**0.000**		worst metrics in RC test
Lin D	*0*.*806*	*0*.*926*	0.970	*0*.*466*	*0*.*425*	*0*.*364*		weak in all tests
