# Supplementary figures and images for: Novel comparison of evaluation metrics for gene ontology classifiers reveals drastic performance differences
Source: PLoS Comput Biol. 2019 Nov 4;15(11):e1007419. doi: 10.1371/journal.pcbi.1007419 (PMC6855565; doi:10.1371/journal.pcbi.1007419)

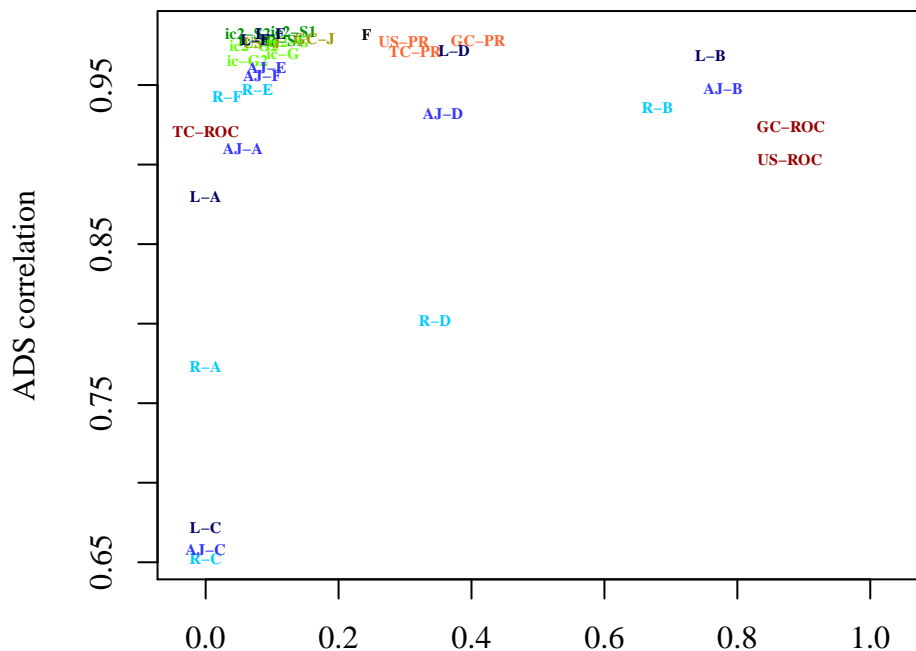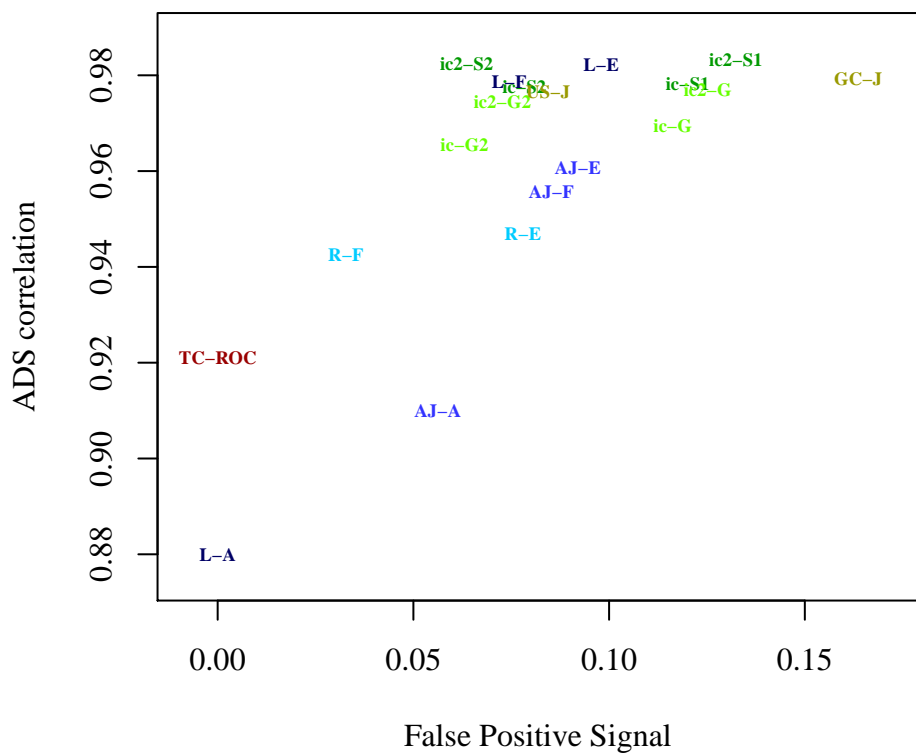

Supplement: S1 Fig — This scatter plot represents EvM performances on MouseFunc data set. Labels and abbreviations, used here, are explained in Fig 8. (PDF) [file pcbi.1007419.s001.pdf]

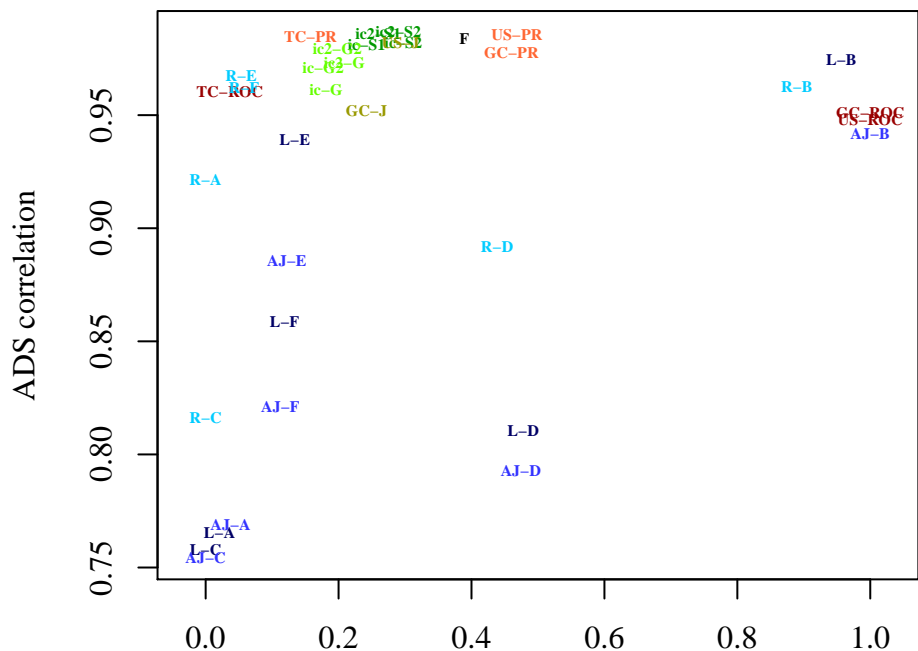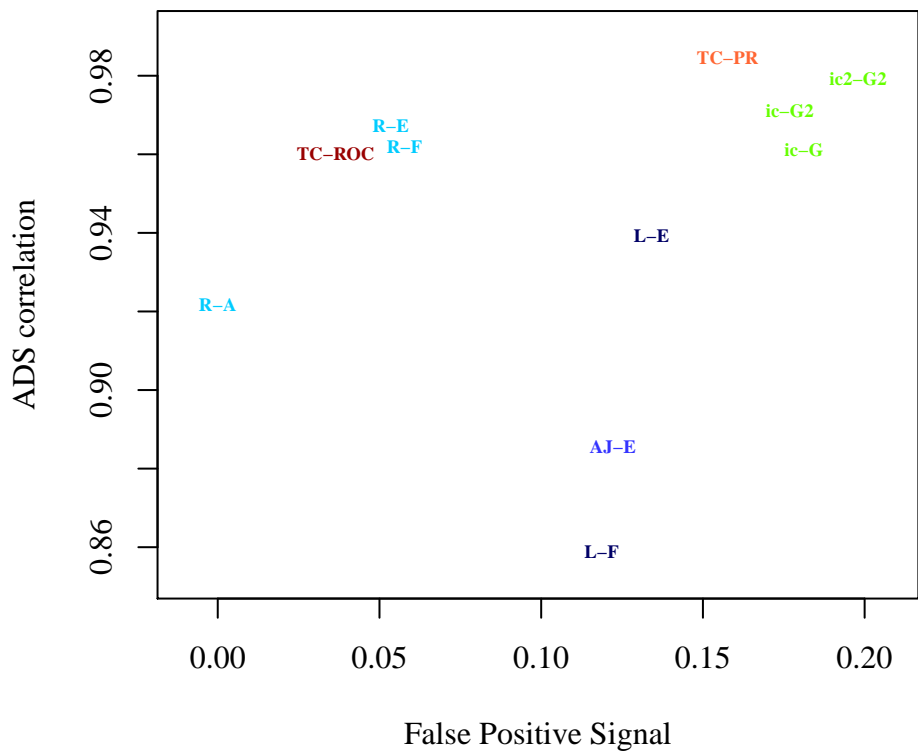

Supplement: S2 Fig — This scatter plot represents EvM performances on Uniprot data set. Labels and abbreviations, used here, are explained in Fig 8. (PDF) [file pcbi.1007419.s002.pdf]

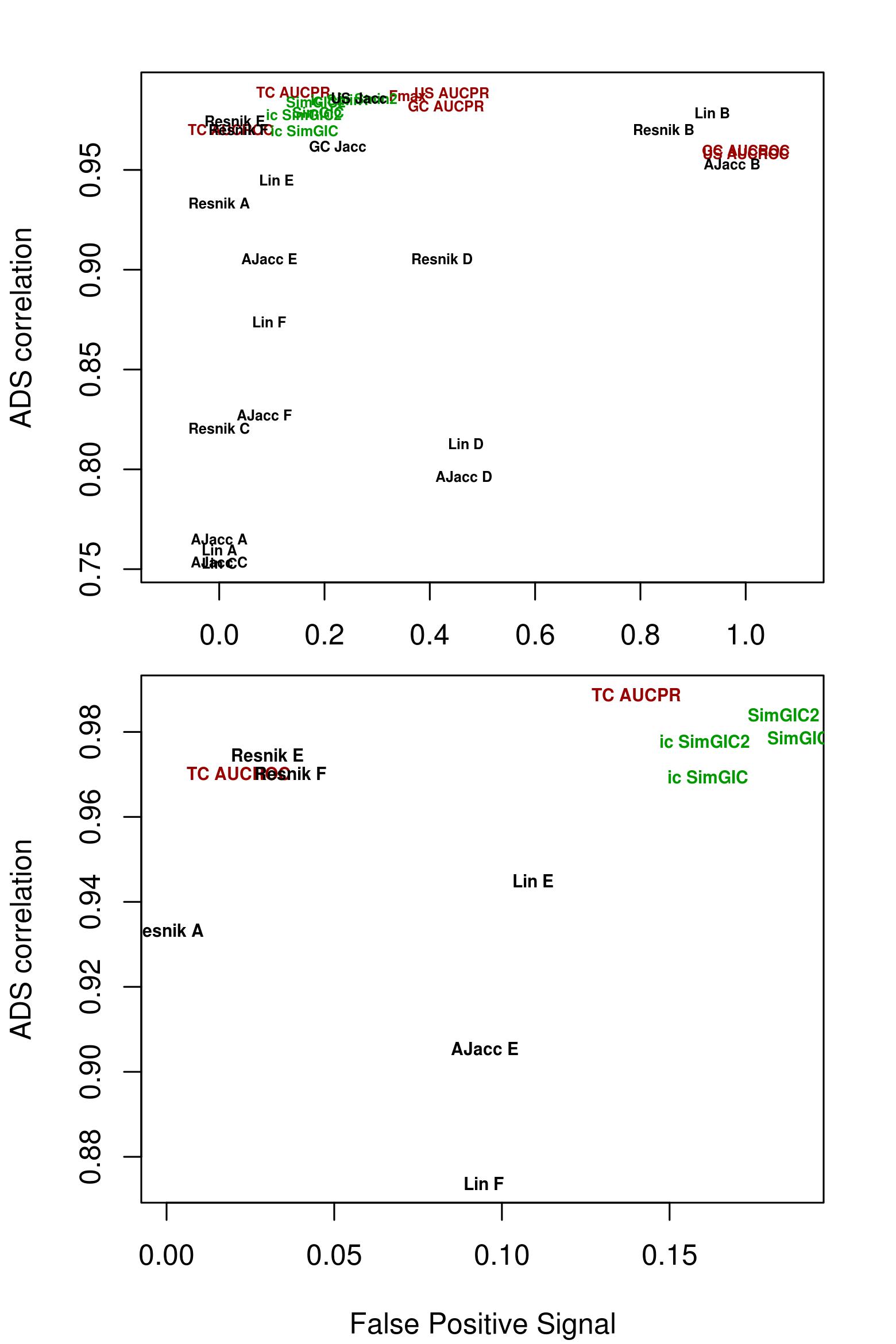

Supplement: S1 File — This compressed archive file represents the evaluation metric scores for all generated AP and FP sets, obtained with k = 2. (GZ) [file pcbi.1007419.s005.tar.gz › res-2019-01-r2/uniprot.1000_scattered_labels.jpeg]

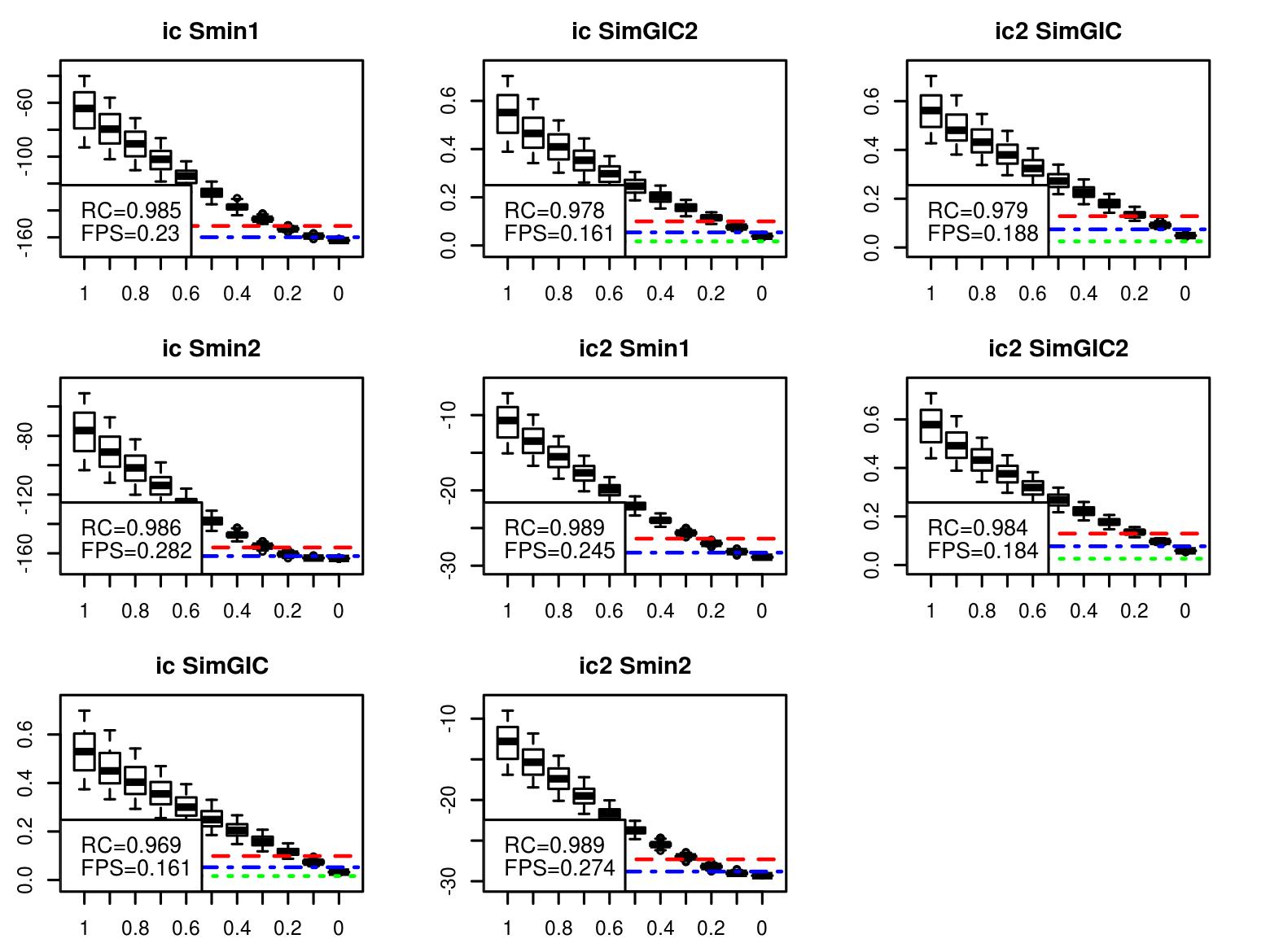

Supplement: S1 File — This compressed archive file represents the evaluation metric scores for all generated AP and FP sets, obtained with k = 2. (GZ) [file pcbi.1007419.s005.tar.gz › res-2019-01-r2/uniprot.1000_boxplot4.jpeg]

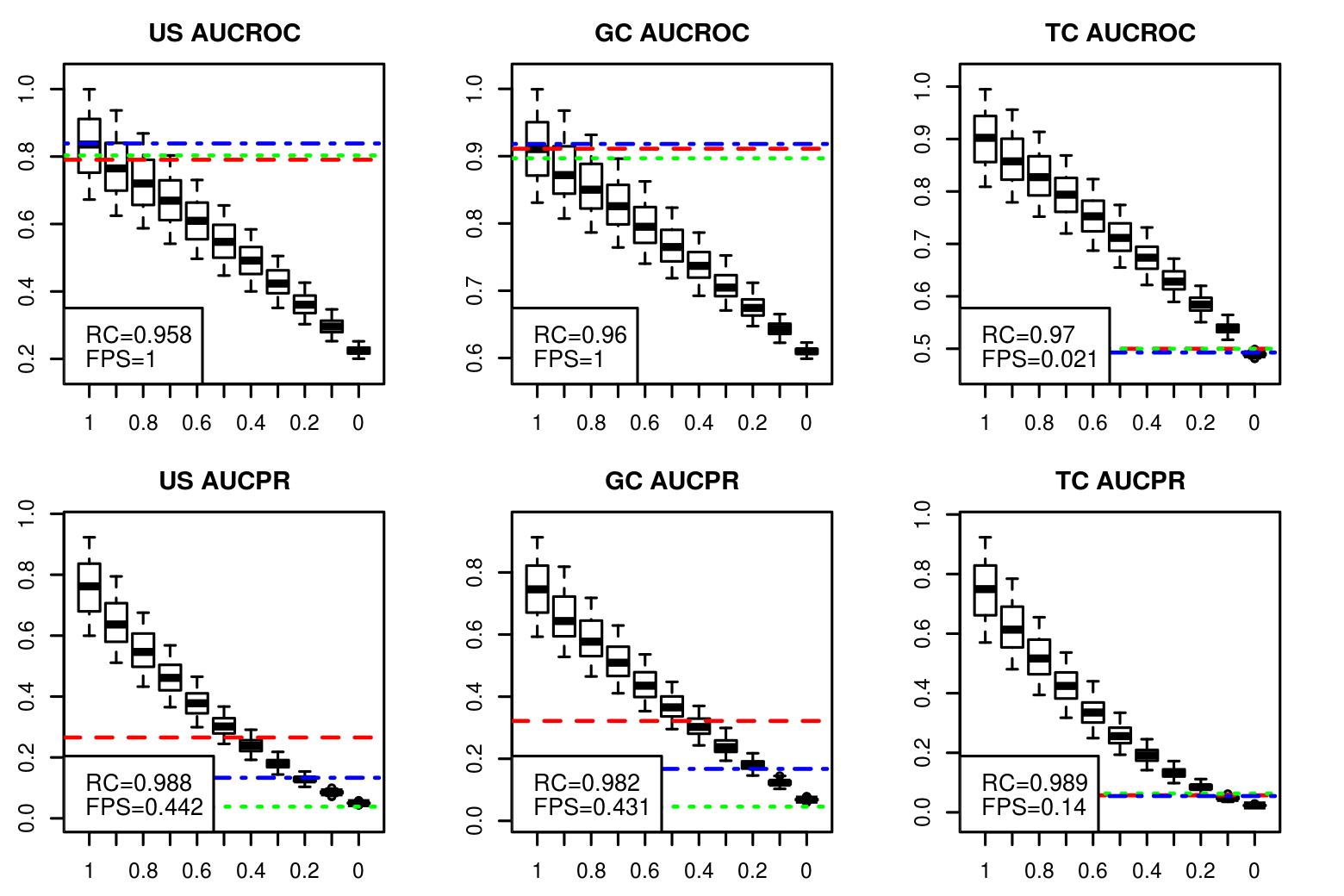

Supplement: S1 File — This compressed archive file represents the evaluation metric scores for all generated AP and FP sets, obtained with k = 2. (GZ) [file pcbi.1007419.s005.tar.gz › res-2019-01-r2/uniprot.1000_boxplot2.jpeg]

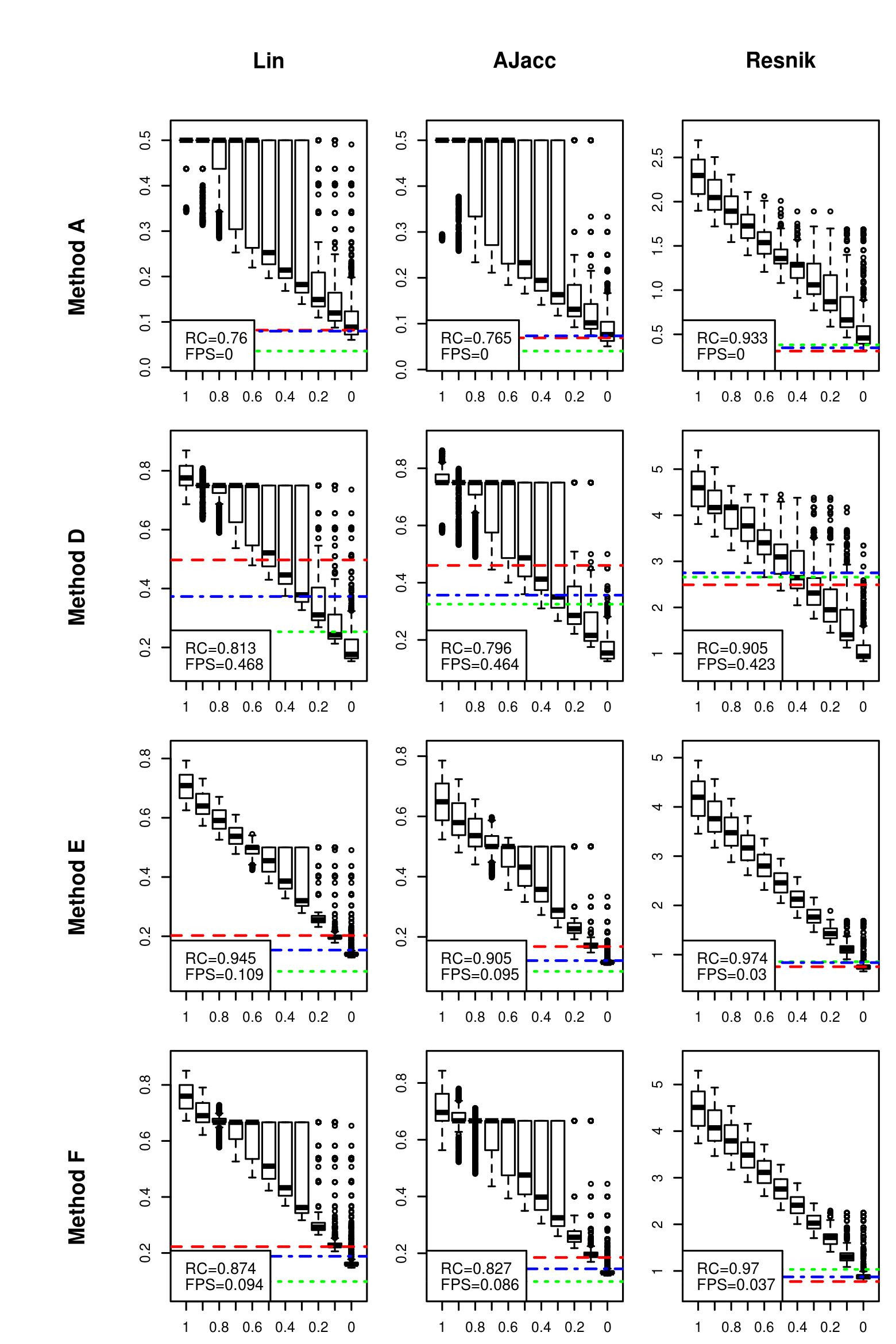

Supplement: S1 File — This compressed archive file represents the evaluation metric scores for all generated AP and FP sets, obtained with k = 2. (GZ) [file pcbi.1007419.s005.tar.gz › res-2019-01-r2/uniprot.1000_boxplot3c.jpeg]

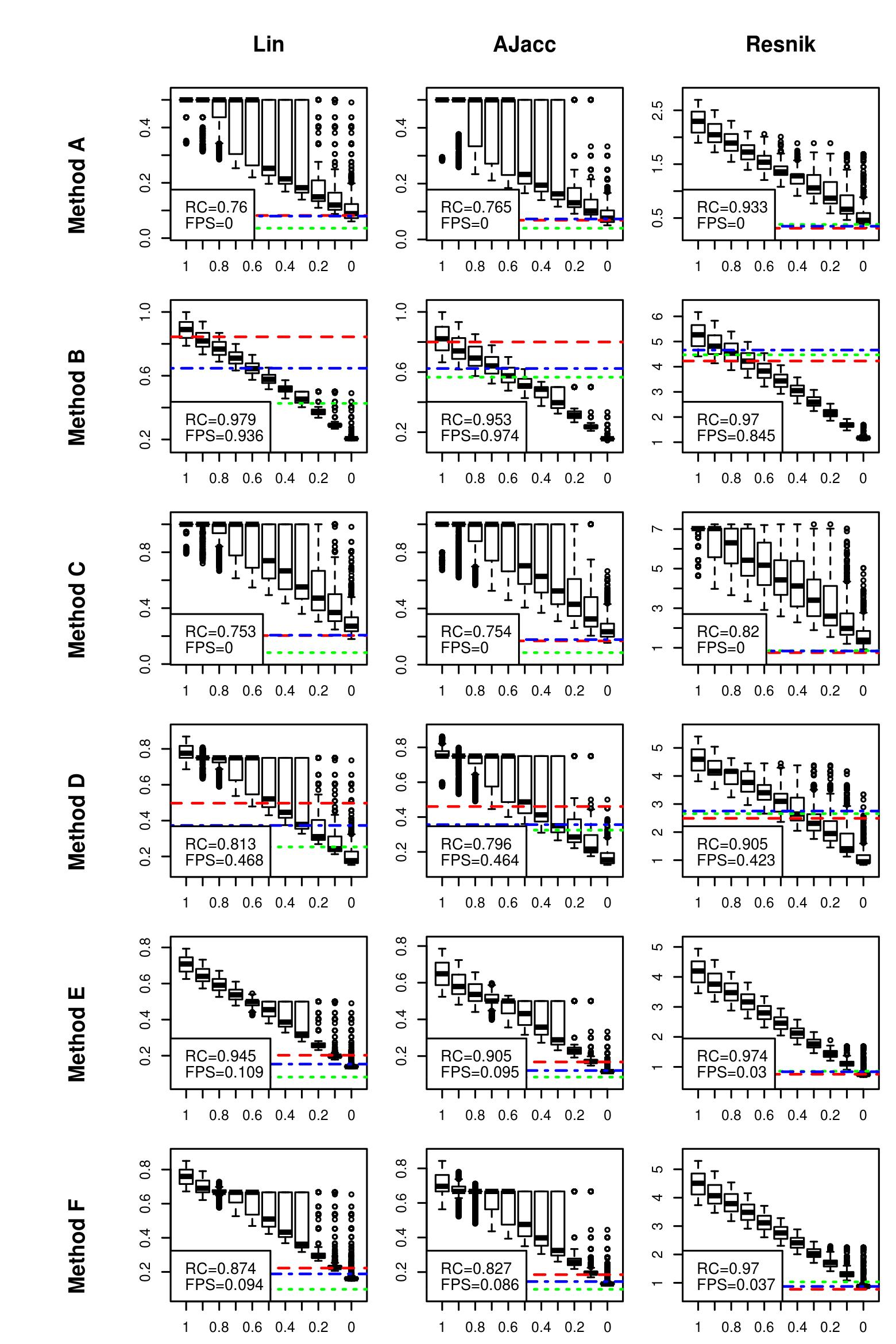

Supplement: S1 File — This compressed archive file represents the evaluation metric scores for all generated AP and FP sets, obtained with k = 2. (GZ) [file pcbi.1007419.s005.tar.gz › res-2019-01-r2/uniprot.1000_boxplot3.jpeg]

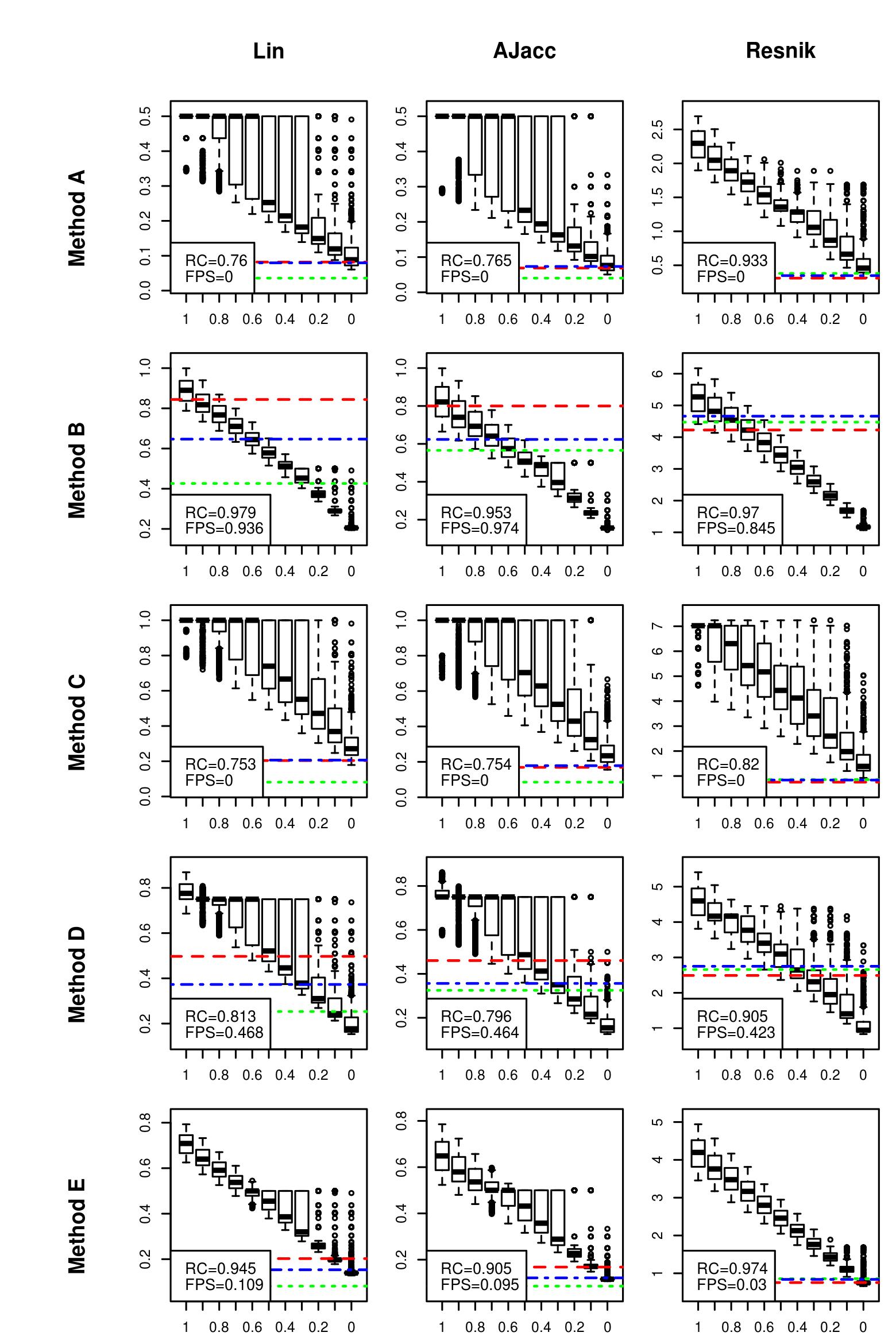

Supplement: S1 File — This compressed archive file represents the evaluation metric scores for all generated AP and FP sets, obtained with k = 2. (GZ) [file pcbi.1007419.s005.tar.gz › res-2019-01-r2/uniprot.1000_boxplot3b.jpeg]

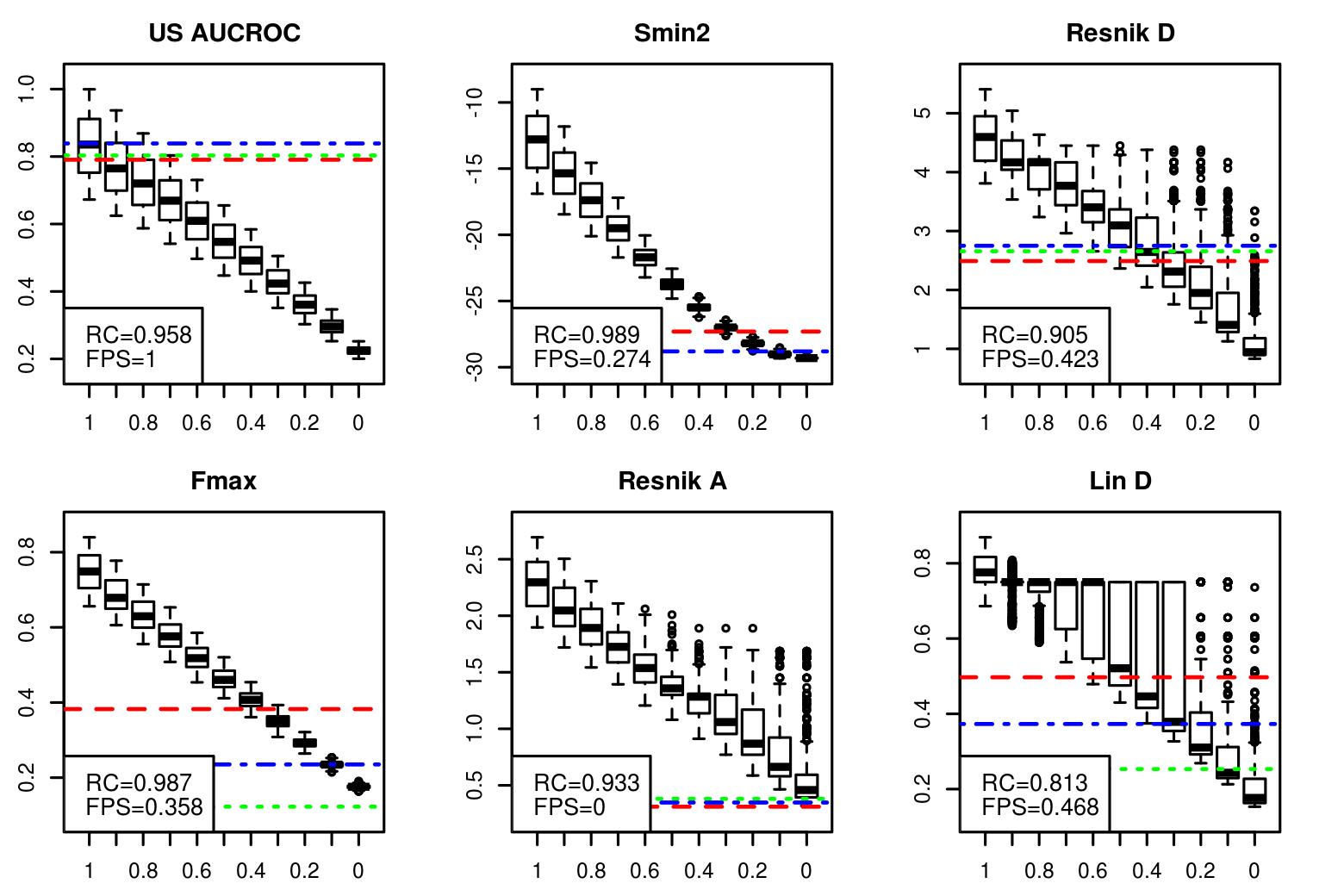

Supplement: S1 File — This compressed archive file represents the evaluation metric scores for all generated AP and FP sets, obtained with k = 2. (GZ) [file pcbi.1007419.s005.tar.gz › res-2019-01-r2/uniprot.1000_boxplot1.jpeg]

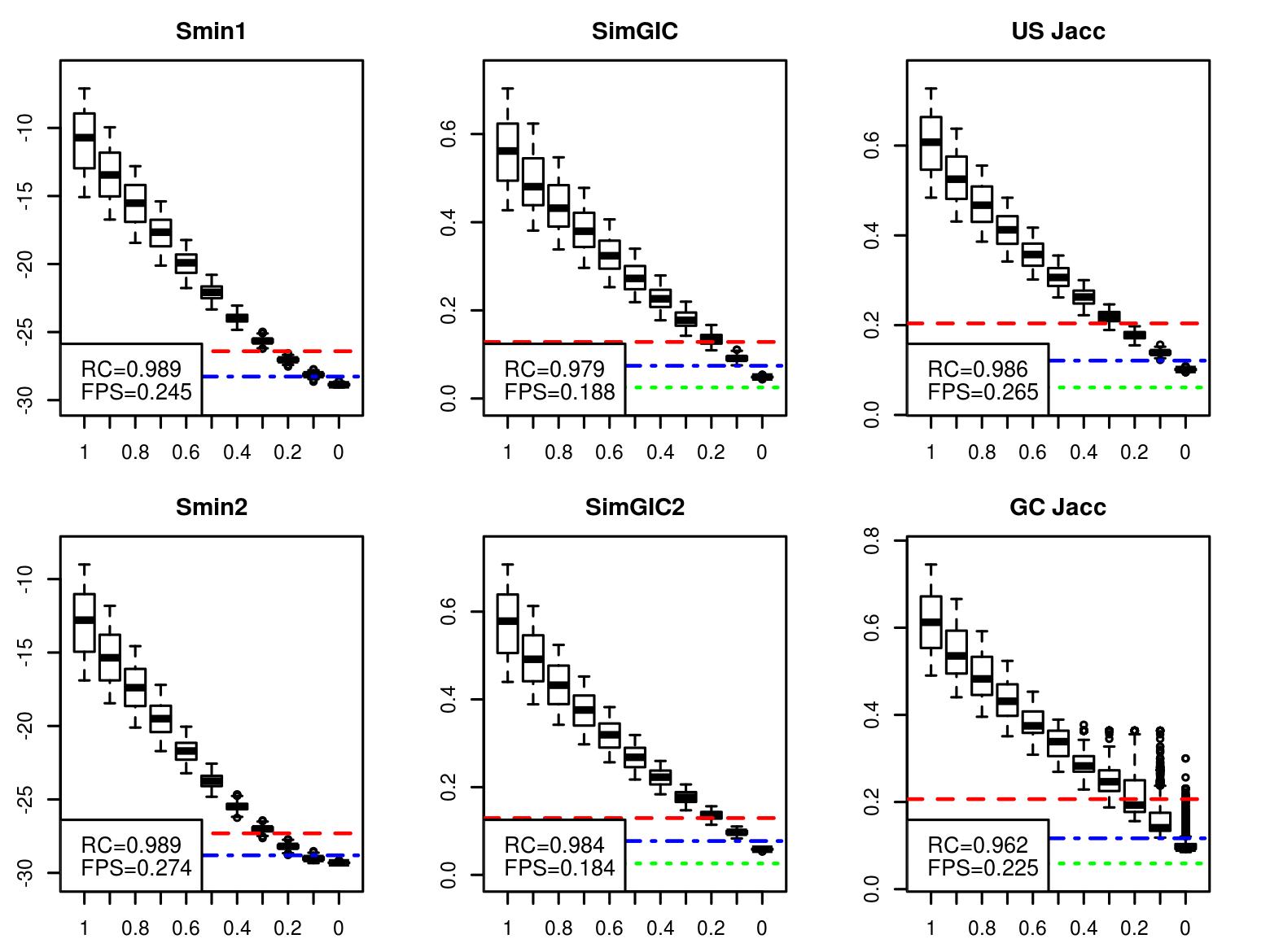

Supplement: S1 File — This compressed archive file represents the evaluation metric scores for all generated AP and FP sets, obtained with k = 2. (GZ) [file pcbi.1007419.s005.tar.gz › res-2019-01-r2/uniprot.1000_boxplot4b.jpeg]

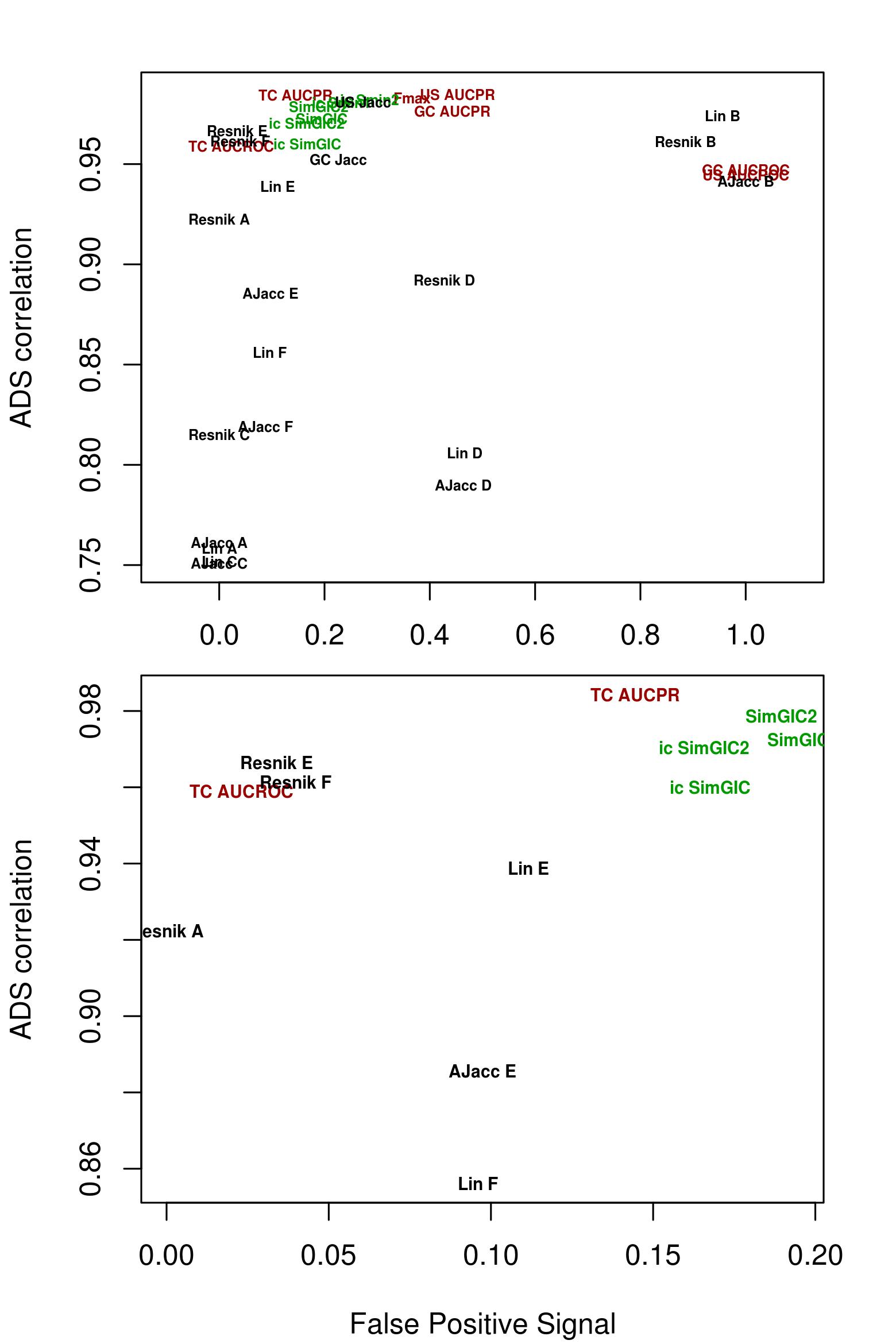

Supplement: S2 File — This compressed archive file represents the evaluation metric scores for all generated AP and FP sets, obtained with k = 3. Our further analysis focuses on this data. (GZ) [file pcbi.1007419.s006.tar.gz › res-2019-01-r3/uniprot.1000_scattered_labels.jpeg]

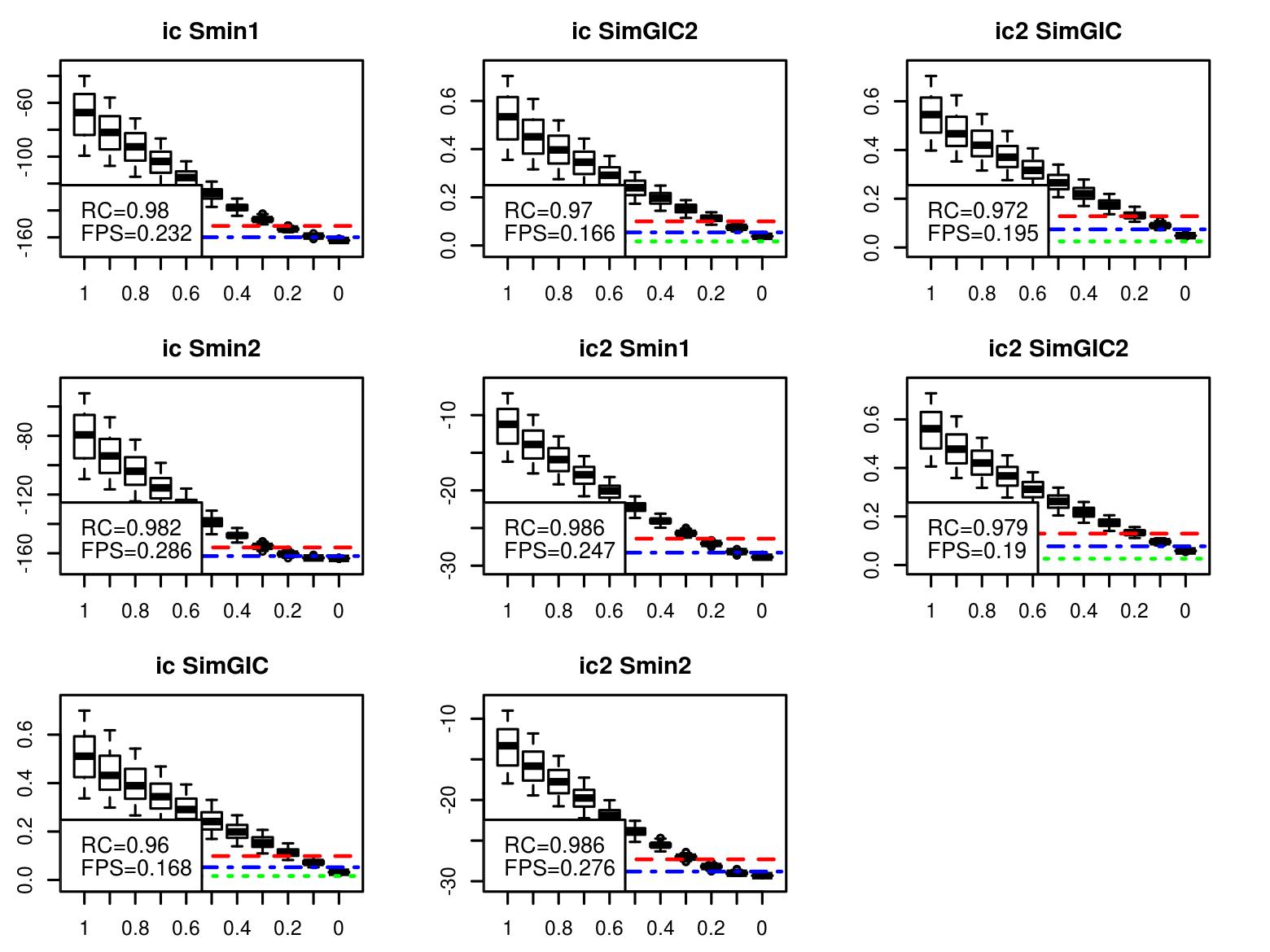

Supplement: S2 File — This compressed archive file represents the evaluation metric scores for all generated AP and FP sets, obtained with k = 3. Our further analysis focuses on this data. (GZ) [file pcbi.1007419.s006.tar.gz › res-2019-01-r3/uniprot.1000_boxplot4.jpeg]

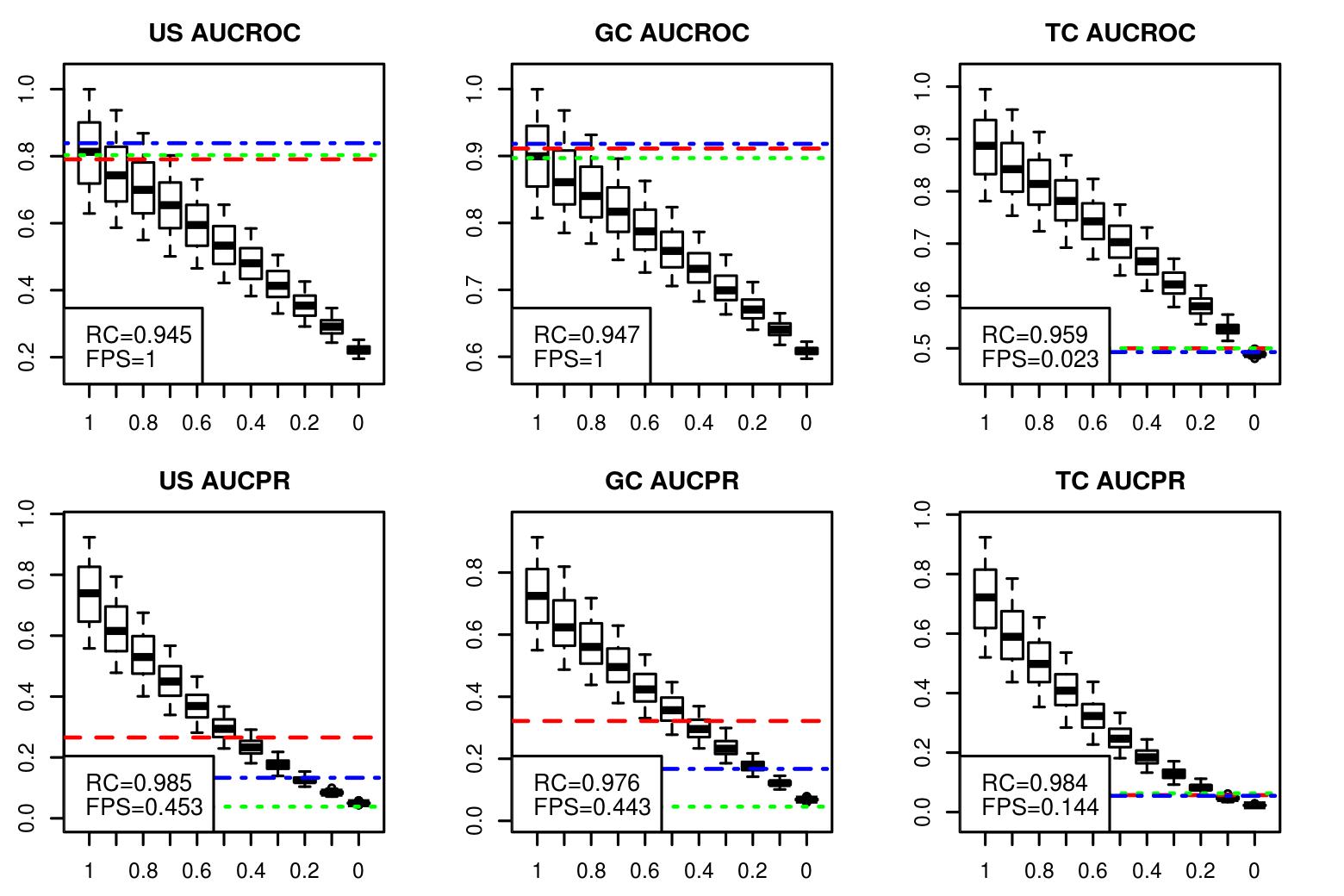

Supplement: S2 File — This compressed archive file represents the evaluation metric scores for all generated AP and FP sets, obtained with k = 3. Our further analysis focuses on this data. (GZ) [file pcbi.1007419.s006.tar.gz › res-2019-01-r3/uniprot.1000_boxplot2.jpeg]

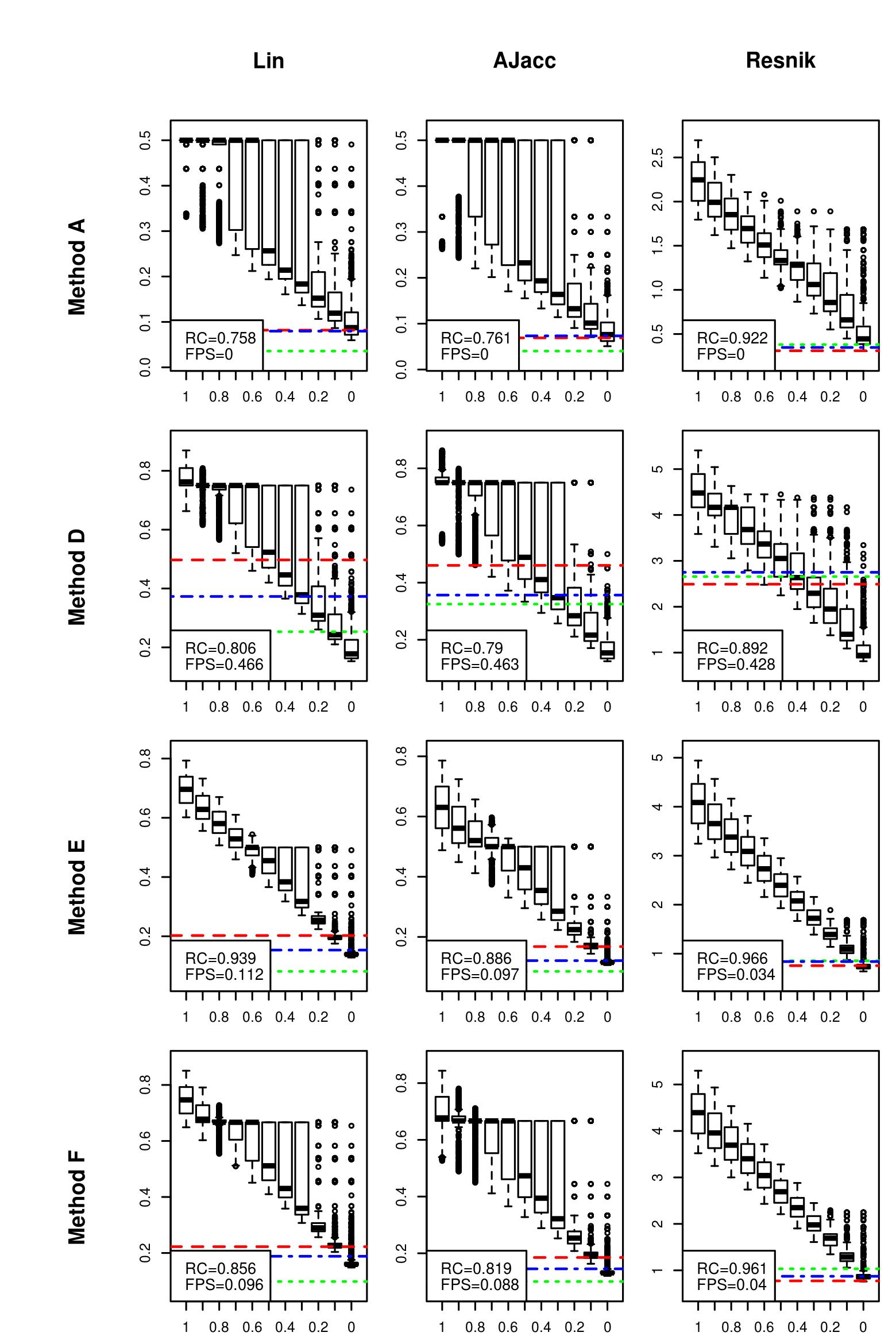

Supplement: S2 File — This compressed archive file represents the evaluation metric scores for all generated AP and FP sets, obtained with k = 3. Our further analysis focuses on this data. (GZ) [file pcbi.1007419.s006.tar.gz › res-2019-01-r3/uniprot.1000_boxplot3c.jpeg]

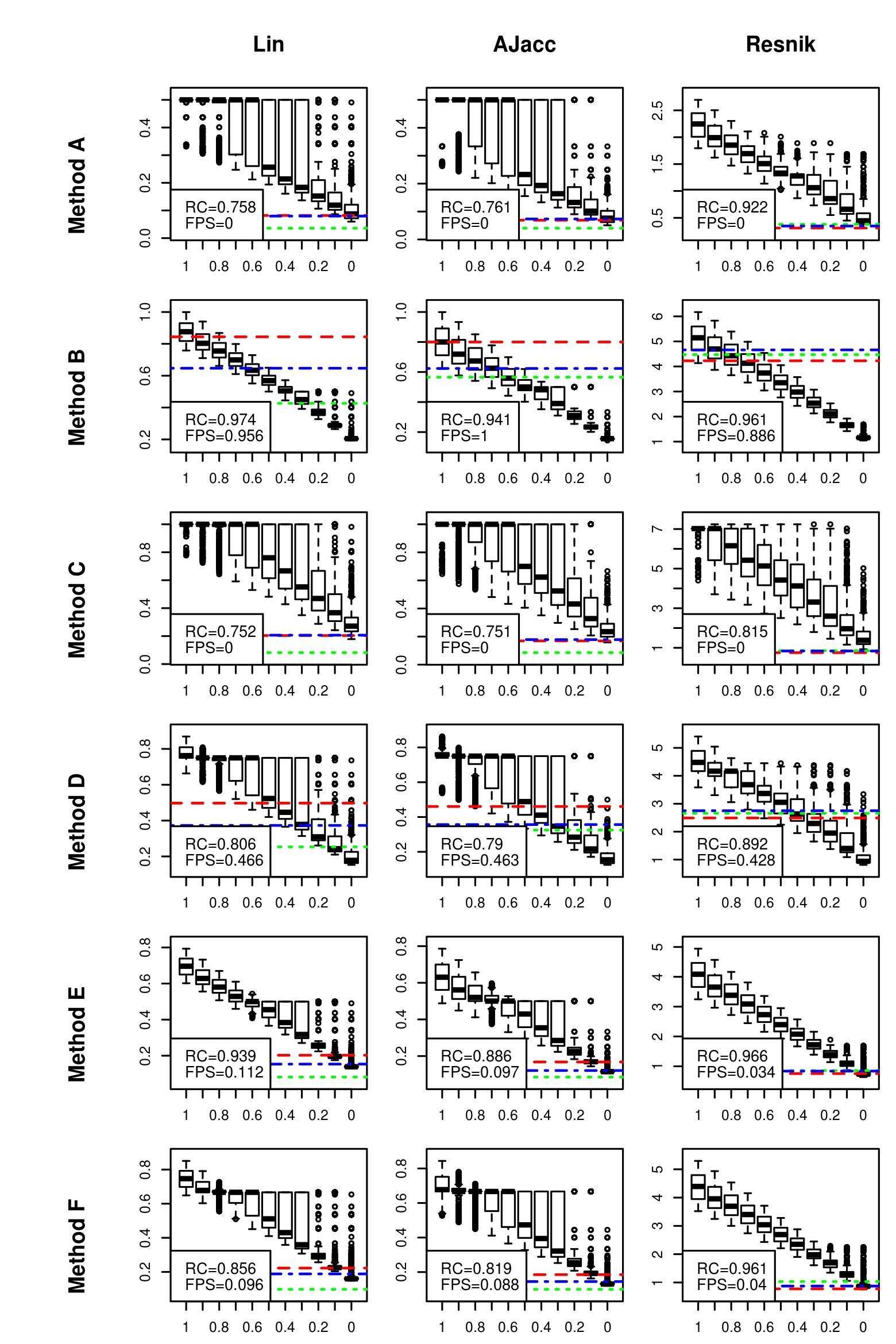

Supplement: S2 File — This compressed archive file represents the evaluation metric scores for all generated AP and FP sets, obtained with k = 3. Our further analysis focuses on this data. (GZ) [file pcbi.1007419.s006.tar.gz › res-2019-01-r3/uniprot.1000_boxplot3.jpeg]

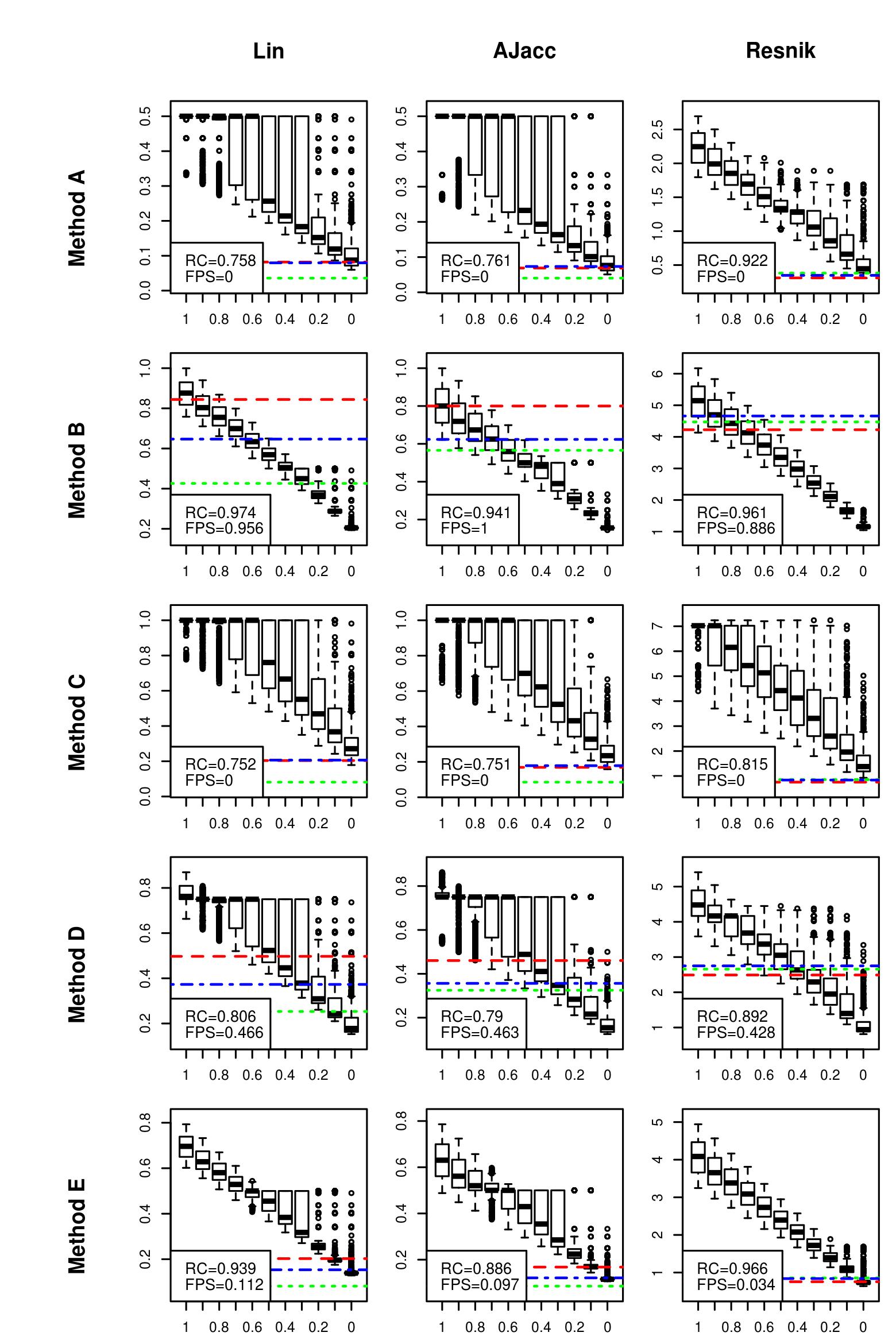

Supplement: S2 File — This compressed archive file represents the evaluation metric scores for all generated AP and FP sets, obtained with k = 3. Our further analysis focuses on this data. (GZ) [file pcbi.1007419.s006.tar.gz › res-2019-01-r3/uniprot.1000_boxplot3b.jpeg]

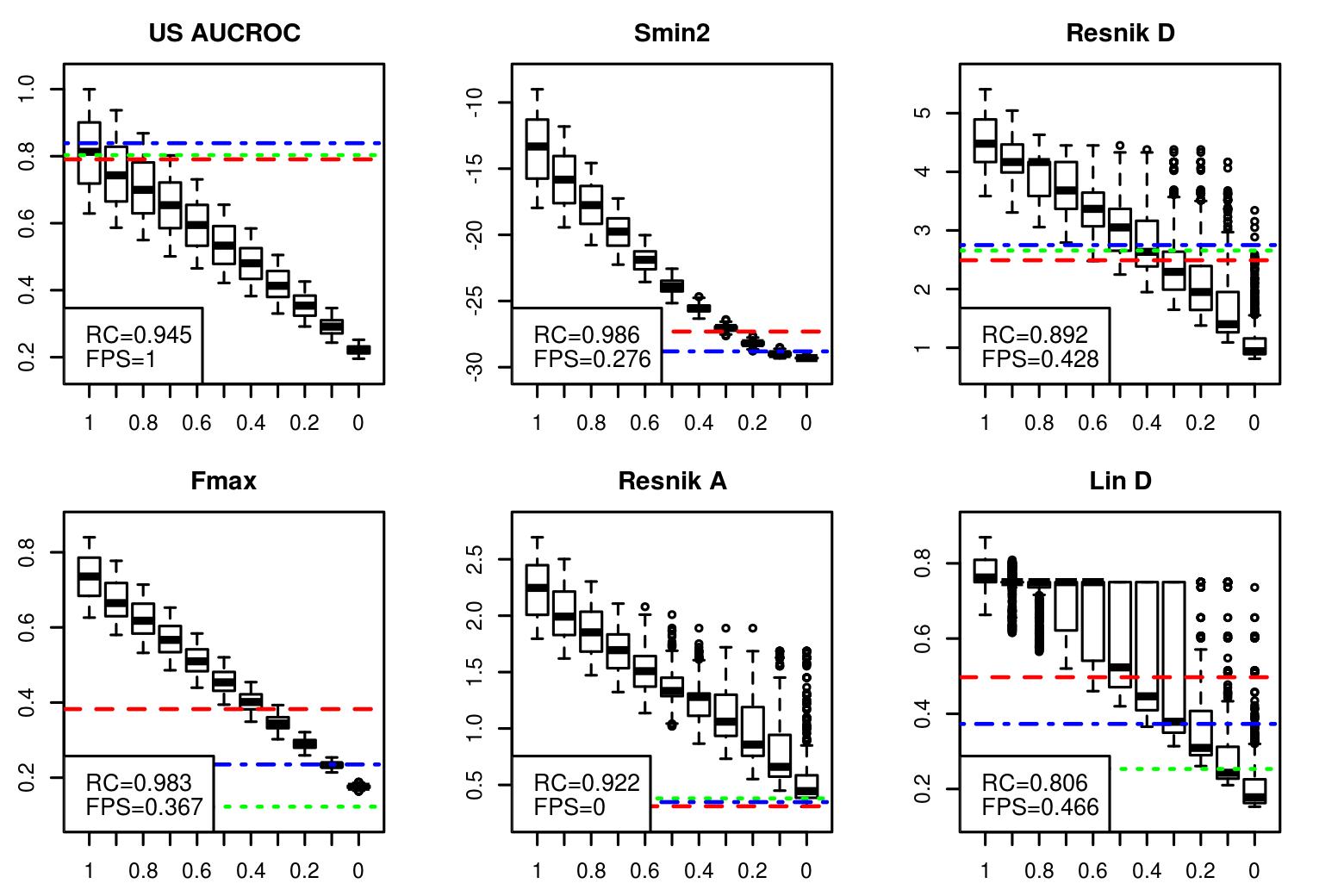

Supplement: S2 File — This compressed archive file represents the evaluation metric scores for all generated AP and FP sets, obtained with k = 3. Our further analysis focuses on this data. (GZ) [file pcbi.1007419.s006.tar.gz › res-2019-01-r3/uniprot.1000_boxplot1.jpeg]

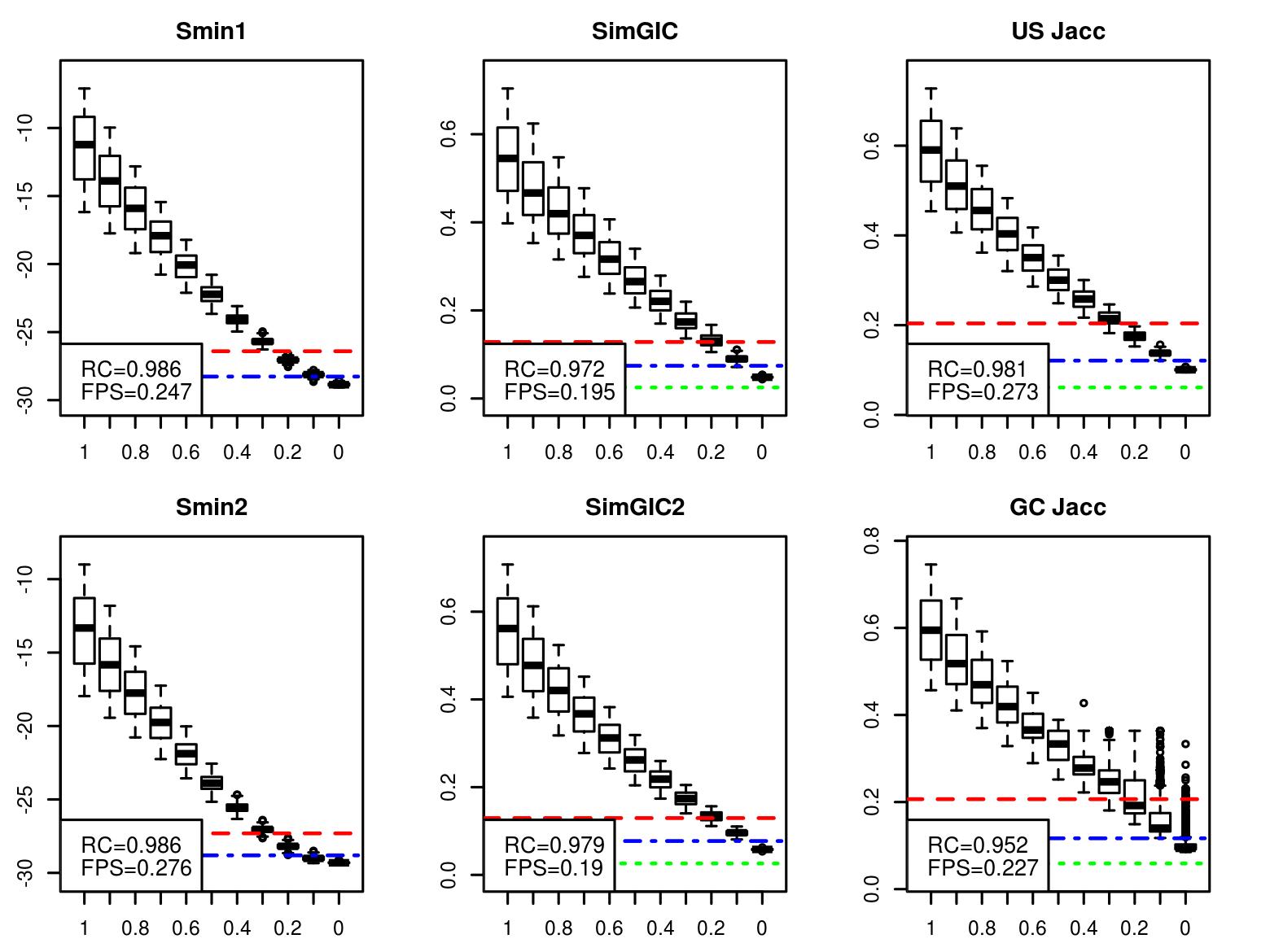

Supplement: S2 File — This compressed archive file represents the evaluation metric scores for all generated AP and FP sets, obtained with k = 3. Our further analysis focuses on this data. (GZ) [file pcbi.1007419.s006.tar.gz › res-2019-01-r3/uniprot.1000_boxplot4b.jpeg]

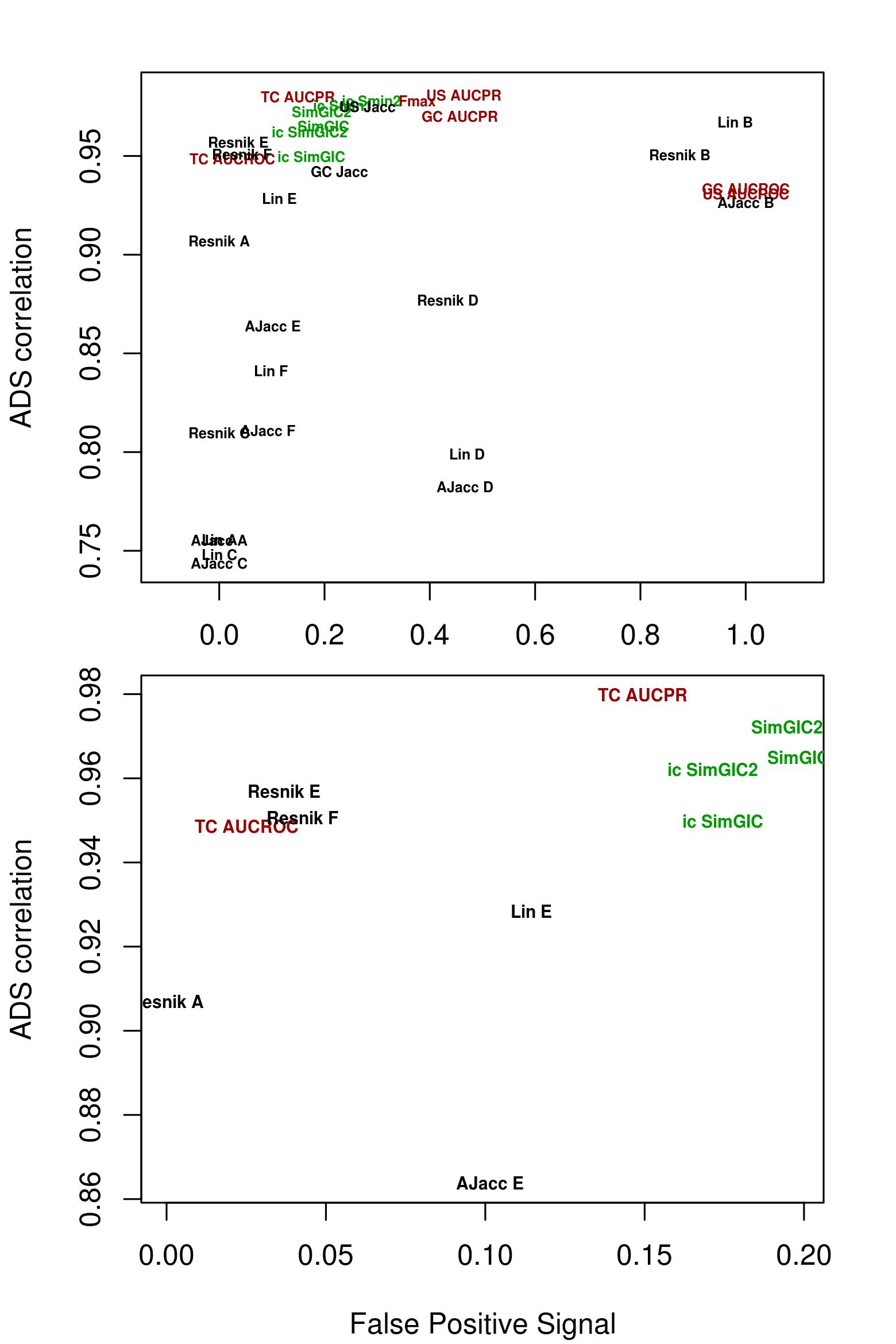

Supplement: S3 File — This compressed archive file represents the evaluation metric scores for all generated AP and FP sets, obtained with k = 4. (GZ) [file pcbi.1007419.s007.tar.gz › res-2019-01-r4/uniprot.1000_scattered_labels.jpeg]

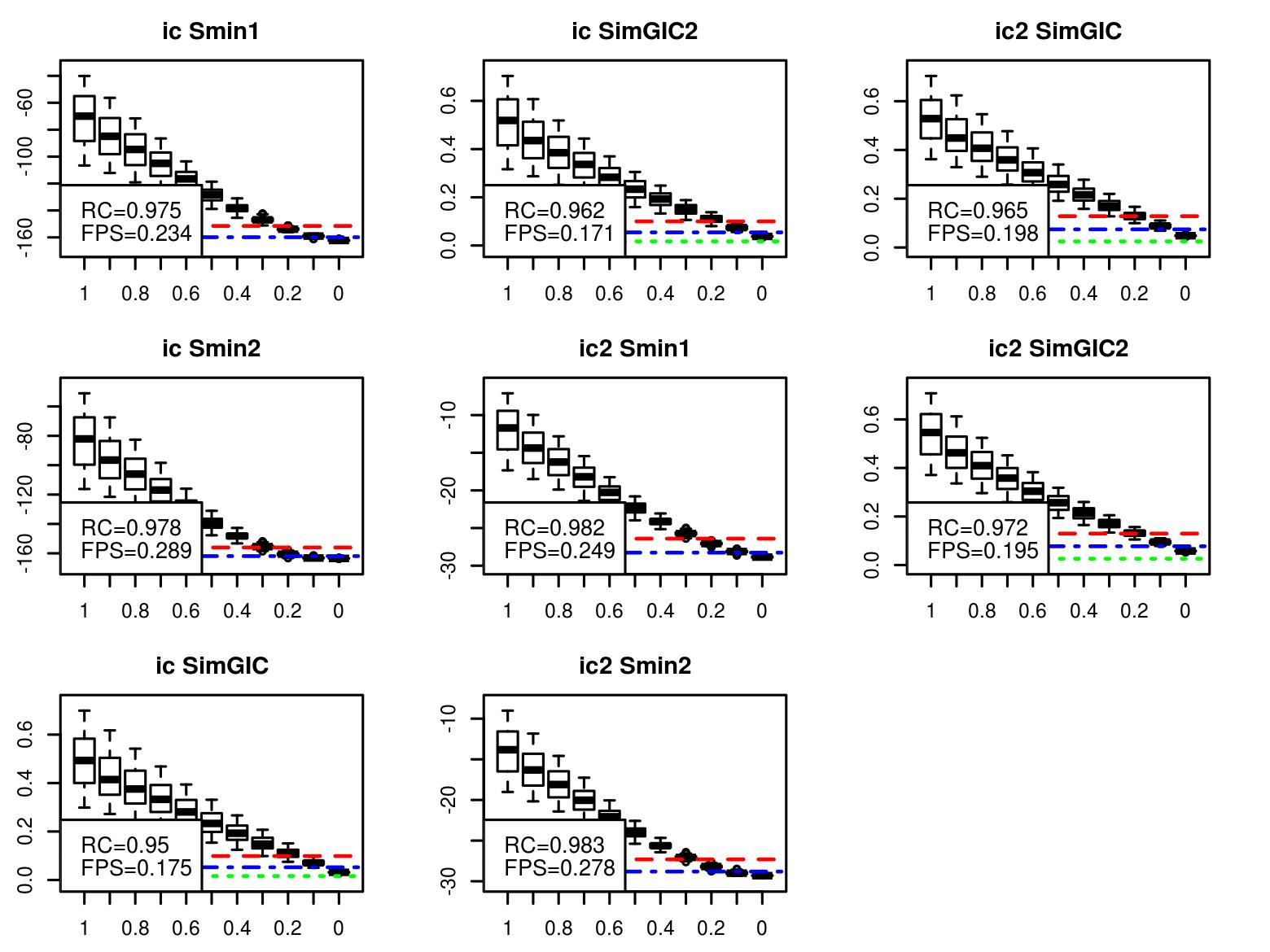

Supplement: S3 File — This compressed archive file represents the evaluation metric scores for all generated AP and FP sets, obtained with k = 4. (GZ) [file pcbi.1007419.s007.tar.gz › res-2019-01-r4/uniprot.1000_boxplot4.jpeg]

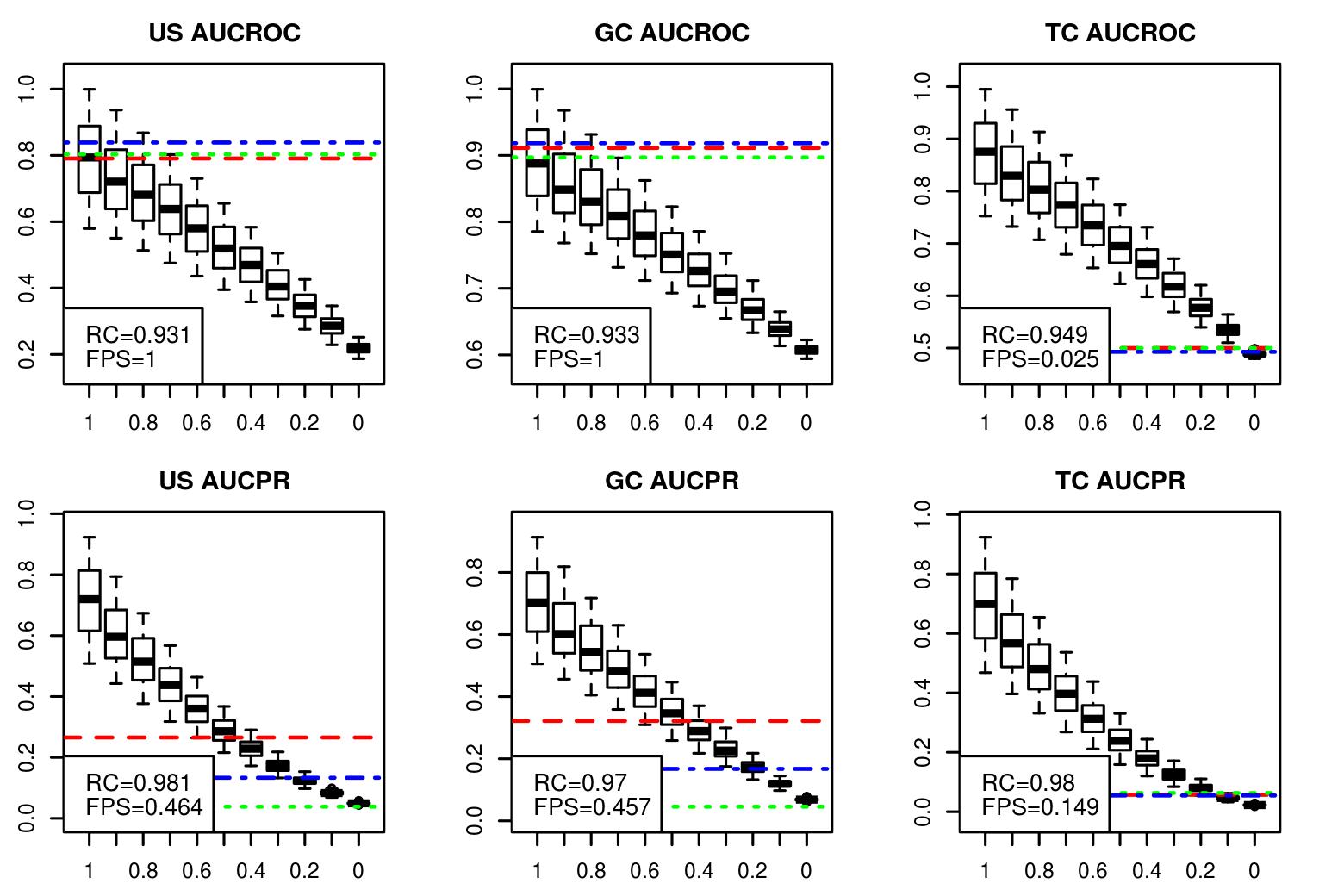

Supplement: S3 File — This compressed archive file represents the evaluation metric scores for all generated AP and FP sets, obtained with k = 4. (GZ) [file pcbi.1007419.s007.tar.gz › res-2019-01-r4/uniprot.1000_boxplot2.jpeg]

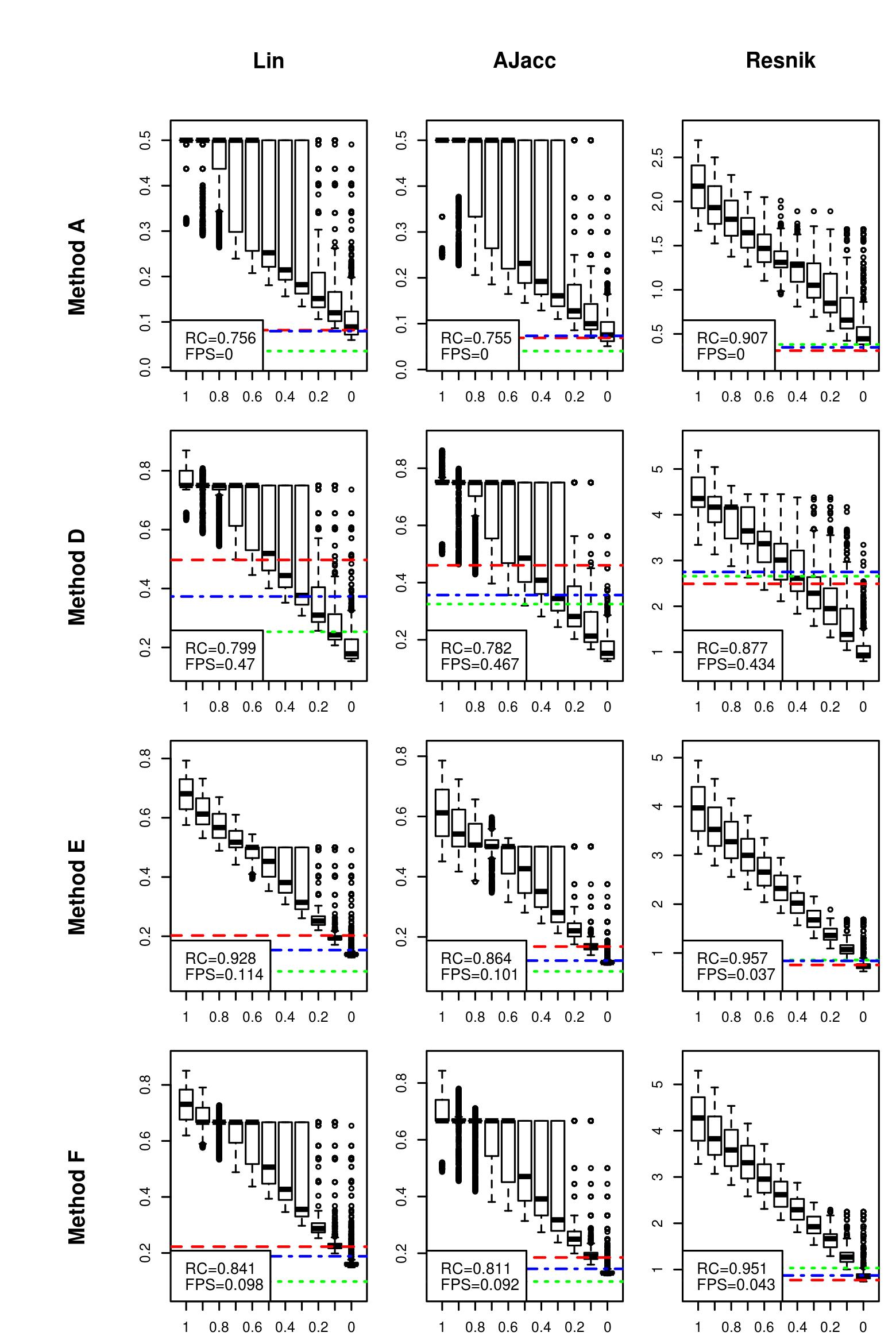

Supplement: S3 File — This compressed archive file represents the evaluation metric scores for all generated AP and FP sets, obtained with k = 4. (GZ) [file pcbi.1007419.s007.tar.gz › res-2019-01-r4/uniprot.1000_boxplot3c.jpeg]

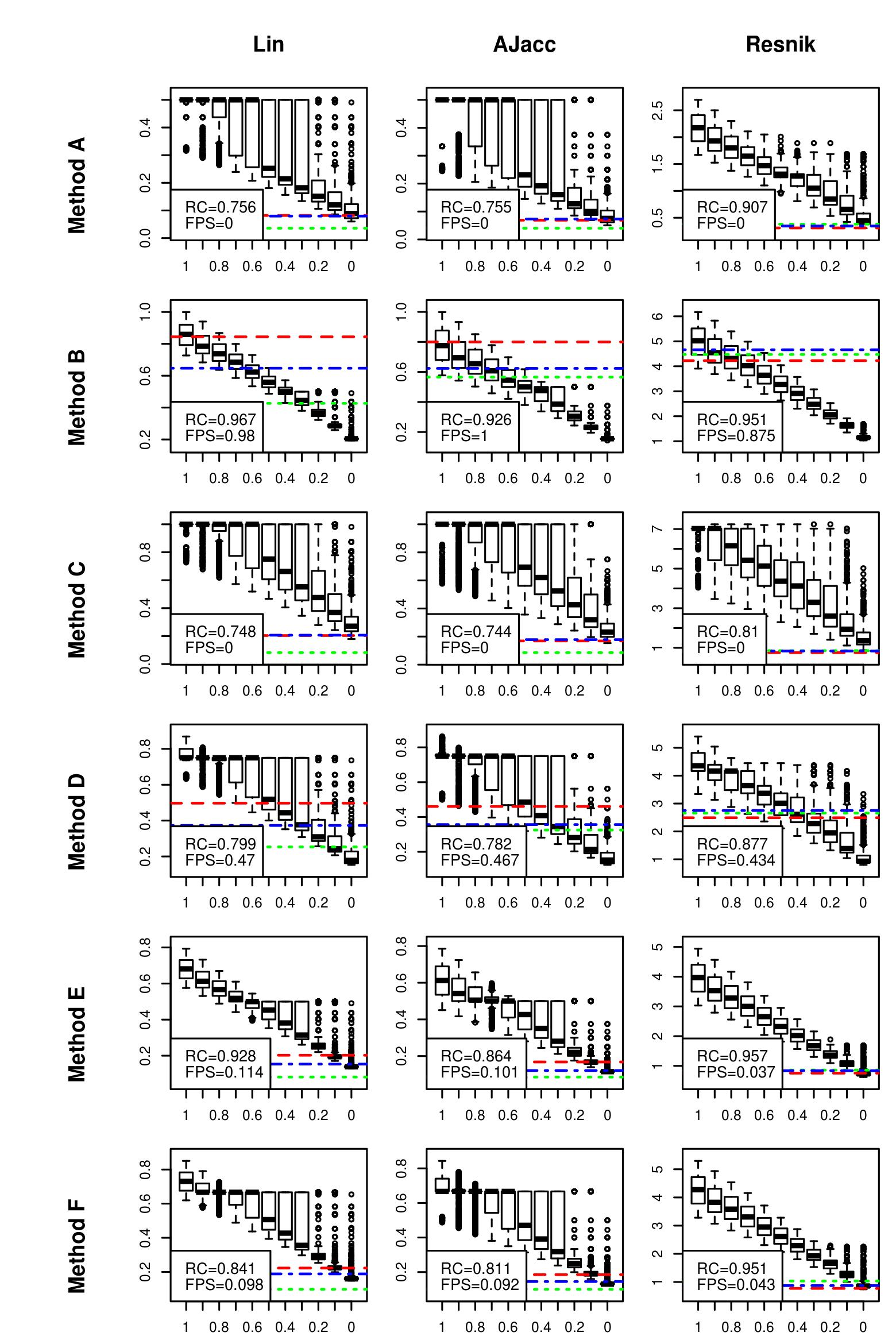

Supplement: S3 File — This compressed archive file represents the evaluation metric scores for all generated AP and FP sets, obtained with k = 4. (GZ) [file pcbi.1007419.s007.tar.gz › res-2019-01-r4/uniprot.1000_boxplot3.jpeg]

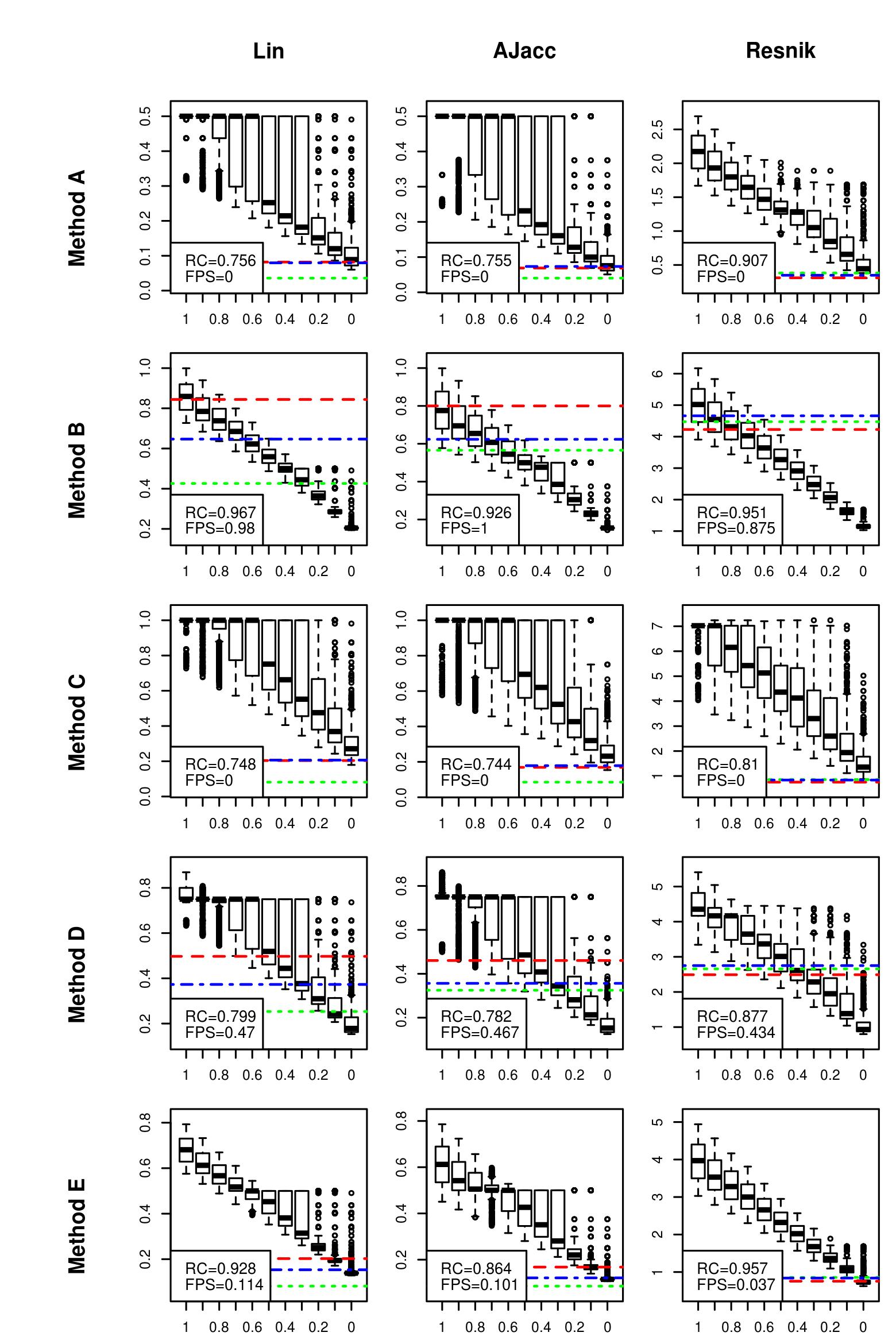

Supplement: S3 File — This compressed archive file represents the evaluation metric scores for all generated AP and FP sets, obtained with k = 4. (GZ) [file pcbi.1007419.s007.tar.gz › res-2019-01-r4/uniprot.1000_boxplot3b.jpeg]

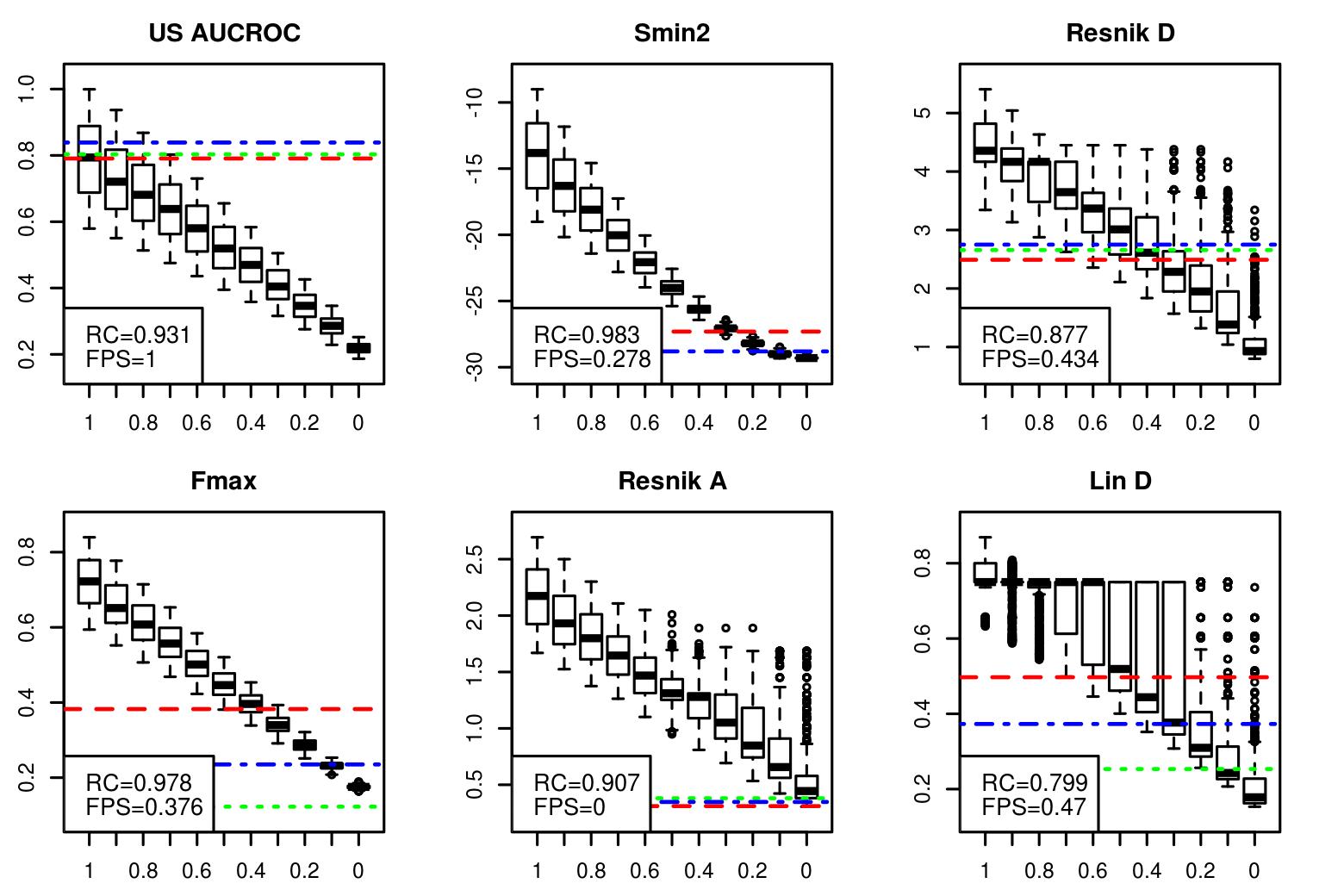

Supplement: S3 File — This compressed archive file represents the evaluation metric scores for all generated AP and FP sets, obtained with k = 4. (GZ) [file pcbi.1007419.s007.tar.gz › res-2019-01-r4/uniprot.1000_boxplot1.jpeg]

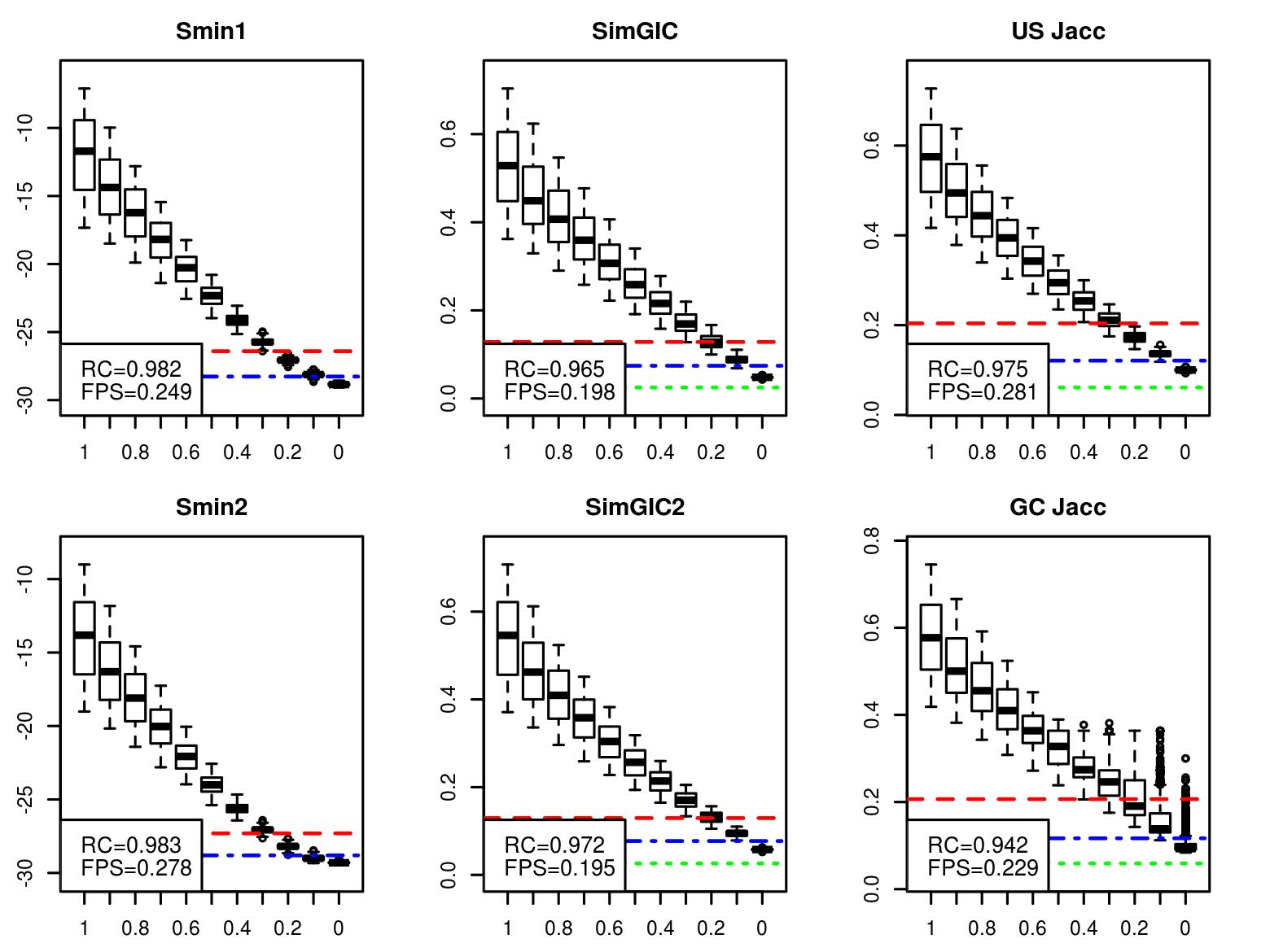

Supplement: S3 File — This compressed archive file represents the evaluation metric scores for all generated AP and FP sets, obtained with k = 4. (GZ) [file pcbi.1007419.s007.tar.gz › res-2019-01-r4/uniprot.1000_boxplot4b.jpeg]
