# Supplementary material for: Novel comparison of evaluation metrics for gene ontology classifiers reveals drastic performance differences
Source: PLoS Comput Biol. 2019 Nov 4;15(11):e1007419. doi: 10.1371/journal.pcbi.1007419 (PMC6855565; doi:10.1371/journal.pcbi.1007419)
Supplement: S1 Text — Pseudocode for generation of ADS. (PDF) [file pcbi.1007419.s003.pdf]

Data: Correct GO predictions, T, for the selected set of genes
Data: Key list representing parent classes for every GO class
parameter: Vector of L Noise Levels, NL
default: $NL = [0, 0.1, 0.2...1]$
parameter: Number of repetitions, K, within each NL
default: $K = 1000$
Result: K by L Matrix, scores with selected Evaluation Metric

```
begin
    Define L as the length of NL ;
    Define output as K by L matrix ;
    Set N_neg = 4 (GO classes per gene in neg. set) ;
    foreach l in [1, 2, .., L] do
        p = NL[l], current noise level ;

        #1. Create positive and negative data
        P_pos = GeneratePosData(T, p) ;
        P_neg = GenerateNegData(T, N_neg) ;

        #2. Create artificial classifier scores to sets
        foreach row in P_pos do
            Select predictor score, s, from Normal(μ = 1, σ = 0.5);
            Add s to the current row;
        end
        foreach row in P_neg do
            Select predictor score, s, from Normal(μ = −1, σ = 0.5);
            Add s to the current row;
        end

        # 3. Combine positive and negative datasets
        P = P_pos ∪ P_neg ;

        # 4. Run Evaluation Metric, EvM
        Output[k, l] = EvM(P, T);
    end
end
```

Algorithm 1: Artificial Dilution Series (ADS) pipeline. Code uses subfunctions *GeneratePosData* and *GenerateNegData*, explained later.

Data: Correct GO predictions, T, for the selected set of genes
Data: Key list representing parent classes for every GO class
parameter: Noise proportion, p
output : Modified GO predictions, P
begin
  Define N_T as the size of T ;
  $P = T$;
  $th_{noise} = round(N_T * p)$, the size threshold for the Noise Set ;

  # **Shifting step**
  Define N_{shift}, a random integer between 0 and N_T;
  Select P_{shift}, a random subset of P of size N_{shift};
  foreach *row in* P_{shift} **do**
    Replace GO class with one of its nearest parents;
  end

  # **Permutation step**
  Define $NoiseSet = []$;
  Define $N_{err} = 0$;
  while $th_{noise} > N_{err}$ **do**
    Select two random rows A and B from $P \setminus NoiseSet$;
    # Genes of these rows will be $gene_A$ and $gene_B$;
    # GO classes of these rows will be GO_A and GO_B ;
    if *IsNoiseClass($gene_A$,GO_B) and IsNoiseClass($gene_B$,GO_A)* **then**
      Swap GO classes between rows A and B in P ;
      Add A and B to $NoiseSet$;
      $N_{err} = N_{err} + 2$;
    end
  end
  return P ;
end

Algorithm 2: GeneratePosData: Generation of positive data with noise proportion p in ADS pipeline. Code uses IsNoiseClass function, explained later.

```
Data: Correct GO predictions, T
parameter: N_neg, number of reported negative GO classes per gene
output    : Negative GO predictions, P_neg
begin
    Define genes(T), the set of unique gene names in T ;
    Define P_neg = [] ;
    foreach gene in genes(T) do
        GO_count = 0 ;
        while GO_count < N_neg do
            pick random GO class, GO_rand, from GO structure ;
            if IsNoiseClass(GO_rand, gene) then
                Add (gene, GO_rand) as item to P_neg ;
                GO_count = GO_count + 1 ;
            end
        end
    end
    Return P_neg ;
end
```

Algorithm 3: GenerateNegData: Generation of negative data in ADS pipeline. Code uses IsNoiseClass function, explained later.

Data: Correct GO predictions, T
Data: Key list representing all ancestor classes for every GO class
Data: Evaluated GO class, GO_{eval}
Data: Evaluated gene, $gene_{eval}$
parameter: Noise threshold, th
default: $th = 0.1$
output : TRUE or FALSE

```
begin
    Get the set of ancestor classes, A_eval, for GO_eval ;
    Define J_max = 0 ;
    foreach row in T do
        Let gene_test be the gene of the current row ;
        if gene_test is gene_eval then
            Let GO_test be the GO class of the current row;
            Get the set of ancestor classes, A_test, for GO_test ;
            Calculate J_test, Jaccard correlation between A_test and A_eval;
            if J_test > J_max then
                J_max = J_test
            end
        end
    end
    if J_max < th then
        Return TRUE ;
    else
        Return FALSE ;
    end
end
```

Algorithm 4: IsNoiseClass. This algorithm tests if GO class qualifies as noise. This is tested against the annotations of the selected gene
