# Supplementary material for: Novel comparison of evaluation metrics for gene ontology classifiers reveals drastic performance differences
Source: PLoS Comput Biol. 2019 Nov 4;15(11):e1007419. doi: 10.1371/journal.pcbi.1007419 (PMC6855565; doi:10.1371/journal.pcbi.1007419)
Supplement: S1 Table — We represent a summary table of all the compared EvMs. Table shows the used abbreviation, core function, used data summary method, used threshold function over the classifier prediction score, used IC weighting and summary method for semantic similarities. In addition we mark the EvMs that have been popular in AFP evaluation, ones that have some novelty and ones that we consider to be simple. We also mark EvMs that we expect to perform badly as negative controls. More detailed description of these EvMs is in suppl. text S2 Text. (PDF) [file pcbi.1007419.s008.pdf]

Type	Abbreviation	Core function	Data summary	Thresh. function	IC weight	SemSim. Sum.	Popular	Neg. Ctrl	Novel	Simple EvM
AUC metrics	US AUC	Area Under ROC Curve	Unstructured	-	-	-		+		+
	GC AUC	Area Under ROC Curve	Gene-Centric	-	-	-				
	TC AUC	Area Under ROC Curve	Term-Centric	-	-	-	+			
	US AUCPR	Area Under Prec-Recall Curve	Unstructured	-	-	-	+			+
	GC AUCPR	Area Under Prec-Recall Curve	Gene-Centric	-	-	-				
	TC AUCPR	Area Under Prec-Recall Curve	Term-Centric	-	-	-				
	Fmax	F-metric	Gene-Centric	max	-	-	+			
group metrics	US Jacc	Jaccard	Unstructured	max	-	-				+
	GC Jacc	Jaccard	Gene-Centric	max	-	-				+
	ic Smin1	Euclidean distance	Unstructured	max	IC1	-				
	ic Smin2	Euclidean distance	Gene-Centric	max	IC1	-			+	
	ic Smin3 (excluded)	Euclidean distance	Unstructured	max	IC1	-			+	
	ic SimGIC	Weighted Jaccard	Gene-Centric	max	IC1	-				
	ic SimGIC2	Weighted Jaccard	Unstructured	max	IC1	-			+	
	ic2 Smin1	Euclidean distance	Unstructured	max	IC2	-	+			
	ic2 Smin2	Euclidean distance	Gene-Centric	max	IC2	-			+	
	ic2 Smin3 (excluded)	Euclidean distance	Gene-CEntric	max	IC2	-			+	
	ic2 SimGIC	Weighted Jaccard	Gene-Centric	max	IC2	-				
	ic2 SimGIC2	Weighted Jaccard	Unstructured	max	IC2	-			+	
Semantic Similarities	Resnik score A	Resnik semantics	Gene-Centric	max	IC1	A				
	Resnik score B	Resnik semantics	Gene-Centric	max	IC1	B		+		
	Resnik score C	Resnik semantics	Gene-Centric	max	IC1	C		+		
	Resnik score D	Resnik semantics	Gene-Centric	max	IC1	D				
	Resnik score E	Resnik semantics	Gene-Centric	max	IC1	E			+	
	Resnik score F	Resnik semantics	Gene-Centric	max	IC1	F			+	
	Lin score A	Lin semantics	Gene-Centric	max	IC1	A				
	Lin score B	Lin semantics	Gene-Centric	max	IC1	B		+		
	Lin score C	Lin semantics	Gene-Centric	max	IC1	C		+		
	Lin score D	Lin semantics	Gene-Centric	max	IC1	D				
	Lin score E	Lin semantics	Gene-Centric	max	IC1	E			+	
	Lin score F	Lin semantics	Gene-Centric	max	IC1	F			+	
	AJacc score A	Jaccard semantics	Gene-Centric	max	-	A				
	AJacc score B	Jaccard semantics	Gene-Centric	max	-	B		+		
	AJacc score C	Jaccard semantics	Gene-Centric	max	-	C		+		
	AJacc score D	Jaccard semantics	Gene-Centric	max	-	D				
	AJacc score E	Jaccard semantics	Gene-Centric	max	-	E			+	
	AJacc score F	Jaccard semantics	Gene-Centric	max	-	F			+	
