# Supplementary material for: Novel comparison of evaluation metrics for gene ontology classifiers reveals drastic performance differences
Source: PLoS Comput Biol. 2019 Nov 4;15(11):e1007419. doi: 10.1371/journal.pcbi.1007419 (PMC6855565; doi:10.1371/journal.pcbi.1007419)
Supplement: S2 Table — Here we evaluate the stability of the results when we vary parameter k and thjacc. Table A shows results for different k values and table B shows results for two values of thjacc. We selected in each comparison one data set, created results for all EvMs using two parameter values and compared the obtained results using Pearson and Spearmann rank correlation. FPS and RC results are evaluated here separately. Tested parameters show very little effect on the results. (PDF) [file pcbi.1007419.s009.pdf]

A

	Compared parameters		Correlation	
	1st K	2nd K	Pearson	rank
RC.uniprot.1000	2	3	0.998587	0.997759
RC.cafa2012	2	3	0.997951	0.998599
RC.mouseFunc	2	3	0.998517	0.998319
RC.uniprot.1000	2	4	0.994401	0.996639
RC.cafa2012	2	4	0.994472	0.996919
RC.mouseFunc	2	4	0.995352	0.996078
FPS.uniprot.1000	2	3	0.999746	1
FPS.cafa2012	2	3	0.99971	0.999368
FPS.mouseFunc	2	3	0.9999	0.997467
FPS.uniprot.1000	2	4	0.999633	0.999507
FPS.cafa2012	2	4	0.999105	0.999438
FPS.mouseFunc	2	4	0.999765	0.993526
		Minimum	0.994401	0.993526
		Maximum	0.9999	1

Comparison of results when K parameter is varied
Comparison is done by calculating correlation between two sets of results
RC and FPS results are evaluated separately

B

	Compared parameters		correlation	
	jacc limit 1	Jacc limit 2	Pearson	Rank
RC.uniprot.1000	0.2	0.8	0.99985	0.99972
RC.cafa2012	0.2	0.8	0.999907	0.998599
RC.mouseFunc	0.2	0.8	0.999198	0.998319
FPS.uniprot.1000	0.2	0.8	0.999664	0.99698
FPS.cafa2012	0.2	0.8	0.999696	0.994526
FPS.mouseFunc	0.2	0.8	0.999608	0.997748
		Minimum	0.999198	0.994526
		Maximum	0.999907	0.99972

Comparison of results when jacc-threshold in noise generation is varied
Comparison is done by calculating correlation between two sets of results
RC and FPS results are evaluated separately
